# Efficacy of repetitive transcranial magnetic stimulation in post-stroke cognitive impairment: an overview of systematic reviews

**DOI:** 10.3389/fneur.2024.1378731

**Published:** 2024-04-22

**Authors:** Linli Zhang, Shan Gao, Chengshuo Wang, Yuanyuan Li, Huateng Yuan, Longjun Cao, Chong Gao

**Affiliations:** ^1^Tianjin Key Laboratory of Exercise Physiology and Sports Medicine, Institute of Sport, Exercise and Health, Tianjin University of Sport, Tianjin, China,; ^2^Department of Rehabilitation Medicine, Haibin People’s Hospital of Tianjin Binhai Newarea, Tianjin, China,; ^3^Beijing Xiaotangshan Hospital, Beijing, China

**Keywords:** post-stroke cognitive impairment, stroke, repetitive transcranial magnetic stimulation, overview of systematic reviews, radar plot

## Abstract

**Objective:**

The reliability of clinical evidence depends on high-quality meta-analyses/ systematic reviews (MAs/SRs). However, there has been no assessment of the quality of MAs/SRs for repetitive transcranial magnetic stimulation (rTMS) in post-stroke cognitive impairment (PSCI), both nationally and internationally. This article seeks to use radar plotting to visually present the quality of MAs/SRs on rTMS for improving cognitive function in PSCI, aiming to offer an intuitive foundation for clinical research.

**Methods:**

Eight Chinese or English databases were systematically searched to collect comprehensive literature, and the retrieval time ranged from inception to 26 March 2024. Literature ranking was calculated using six dimensions: publication year, design type, AMSTAR-2 score, PRISMA score, publication bias, and homogeneity. Finally, radar plots were drafted to present a multivariate literature evaluation. The GRADE tool assessed the strength of evidence for the outcome indicators included in the MAs/SRs.

**Results:**

The 17 articles included had average scores of 12.29, 17, 9.88, 9.71, 12.88, and 12.76 for each dimension. The radar plot showed that an article published in 2023 had the highest rank and a large radar plot area, while an article published in 2021 had the lowest rank and a small radar plot area. The GRADE tool evaluation revealed that 51 pieces of evidence were of very low quality, 67 were of low quality, 12 were of moderate quality, and only one was of high quality.

**Conclusion:**

The average rank score of literature ranged from 8.50 to 17, with higher rankings indicating greater significance in literature reference. Variations in literature quality were attributed to inadequate study planning, irregular literature search and screening, insufficient description of inclusion criteria for studies, and inadequate consideration of bias risk in the included studies. Most MAs/SRs indicated that rTMS was more effective than the control group in enhancing the global cognitive function and activities of daily living in PSCI patients. However, the overall quality of the literature was generally low and needs validation from future high-quality evidence.

**Systematic review registration:**https://www.crd.york.ac.uk/prospero/, identifier CRD42023491280.

## 1 Introduction

Post-stroke cognitive impairment (PSCI) refers to the clinical symptoms where cognitive function is affected to varying degrees after a stroke and lasts up to 6 months (1, 2). According to a survey, it was found that between 17 and 92% of stroke patients experience cognitive dysfunction 3 months after the onset of the condition, which has a significant negative impact on the life quality and activities of daily living (ADL) of patients (3). Patients with PSCI exhibit complex clinical symptoms due to the etiology of the disease, the location and size of the lesion, and potential complications. Typical symptoms include attention deficits, memory impairment, and learning difficulties (4). PSCI has an impact on the communication and daily life abilities of patients. It also decreases their compliance and motivation for rehabilitation and can progress into dementia as the disease worsens. This significantly hinders the rehabilitation process and prevents patients from rejoining their families and society (5).

Comprehensive strategies to address PSCI involve managing established risk factors, utilizing pharmacotherapy, and participating in rehabilitation (6). The primary approaches to prevent PSCI consist of reducing the risk factors for stroke, averting the onset of stroke, and implementing secondary stroke prevention measures (7). Hypertension, hyperglycemia, and hyperlipidemia are common risk factors for cognitive impairment, actively controlling which can effectively reduce the risk of cognitive decline (8). There is still a lack of consistent guidelines internationally regarding drug treatment for PSCI, and the currently commonly used drugs often cause various adverse reactions, such as gastrointestinal disorders (9). Traditional Chinese rehabilitation methods, such as herbal and acupuncture treatments, have not shown significant success in treating cognitive impairment (10). Additionally, cognitive function training, including general cognitive training and enriched rehabilitation training, is time-consuming, tedious, and often leads to poor patient compliance, posing challenges in achieving the desired outcomes (11). Contemporary rehabilitation treatments frequently include neuromodulation and artificial intelligence technologies. While studies have shown that artificial intelligence technologies such as computerized training, virtual reality, and rehabilitation robots can effectively improve the cognitive function of patients with PSCI, they are not widely used in clinical practice due to the high equipment costs (12, 13). Neuromodulation techniques like transcranial direct current stimulation (tDCS) and repetitive transcranial magnetic stimulation (rTMS) are frequently employed to improve cognitive function in both clinical and experimental settings nowadays (14, 15).

rTMS is a non-invasive neuromodulation technique with the advantages of high safety, few side effects, and simple operation (16). rTMS generates induced currents by pulsed magnetic fields acting on the cerebral cortex, altering the membrane potential of nerve cells, thereby affecting the metabolism of substances in the brain and triggering a series of physiological and biochemical responses (17). Based on the varying frequencies of stimulation, it is categorized into low-frequency rTMS (≤1 Hz) and high-frequency rTMS (>1 Hz). Low-frequency rTMS reduces the excitability of the cerebral cortex and functions in an inhibitory capacity. In contrast, high-frequency rTMS raises the excitability of the cerebral cortex and serves an excitatory function. Repeated superimposed pulsed stimulation leads to a cumulative effect, which can impact both localized and distal regions, resulting in long-term inhibitory and excitatory effects (18). The stimulation pulse can be classified into single-pulse, double-pulse, and repetitive transcranial magnetic stimulation. Additionally, rTMS comprises two distinct stimulation modes: intermittent theta burst stimulation (iTBS) and continuous theta burst stimulation (cTBS). In recent years, there has been much focus on the effectiveness of rTMS in PSCI. Many studies have confirmed its clinical effectiveness, showing improvements in global cognitive function, executive function, and activities of daily living (ADL) (14, 19). Currently, Numerous articles have been published on meta-analyses/systematic reviews discussing the effectiveness of rTMS for post-stroke cognitive impairment. However, the consistency of intervention methods, outcome indicators, and conclusions in these articles was low, and the quality varied. Low-quality MAs/SRs may mislead and hinder research development to a certain extent, and evaluating published MAs/SRs is vital to promote the development of rTMS in the field of PSCI. Currently, relevant studies have yet to be published in this area.

The overview of systematic review is a method for a comprehensive study of evidence from multiple relevant MAs/SRs. Compared to MAs/SRs, it can provide higher quality evidence (20). The article utilizes radar plots to present data in six dimensions: qualitative evaluation of publication year, design type, homogeneity, publication bias, and quantitative assessment using the AMSTAR-2 methodological quality score, PRISMA report quality score (21, 22). Additionally, the article utilized the GRADE tool to assess the strength of evidence in MAs/SRs outcome indicators, aiming to analyze the quality of the literature qualitatively and quantitatively with the assistance of a radar plot and the GRADE tool. This approach provides a visual foundation for decision-making in clinical settings.

## 2 Materials and methods

### 2.1 Study enrollment and reporting

Our study (CRD42023491280) has been officially registered in the International Prospective Register of Systematic Reviews, PROSPERO. Although the reporting guideline for overviews is currently in progress, we have presented our findings using the Preferred Reporting Items for Meta-Analyses and Systematic Reviews (PRISMA) 2020 (23, 24).

### 2.2 Inclusion criteria

#### 2.2.1 Study design

Chinese and English meta-analyses/systematic reviews based on randomized controlled trials (RCTs) investigating the effectiveness of rTMS for PSCI were incorporated into the study.

#### 2.2.2 Subjects

The study subjects were patients with PSCI (there is no clear diagnostic gold standard, but for reference, a definite stroke event and cognitive impairment within 6 months of the stroke event) of any age and gender.

#### 2.2.3 Interventions

In the experimental group, rTMS was administered alone or in combination with other treatments such as routine treatment, cognition training, acupuncture, rehabilitation therapy, computer-assisted cognitive rehabilitation, pharmacotherapy, hyperbaric oxygen, and occupational therapy. The control group received sham-rTMS, other treatments, or sham-rTMS combined with other therapies. There were no restrictions on the parameters of the stimulation site, stimulation frequency, and stimulation time of rTMS.

#### 2.2.4 Outcome indicators

The primary outcome indicators include (1) global cognitive function measured by Montreal Cognitive Assessment (MOCA), Mini-mental State Examination (MMSE), or Alzheimer’s disease assessment scale-cognitive subscale (ADAS-Cog); (2) other cognition performance: (a) attention measured by Auditory Continuous Performance Test (Auditory-CPT) or Visual-CPT; (b) executive function measured by Word of Color Word Test, Color of Color Word Test, Tower of London test, Victoria Stroop Test-time (VST-time), Victoria Stroop Test-error words (VST-error words), Loewenstein Occupational Therapy of Cognitive Assessment (LOTCA), Stroop Color and Word Test-C section (SCWT-Cs), or Stroop Color and Word Test-Consuming (SCWT-C); (c) working memory measured by Digit Symbol Test (DST), Digital Span Test (DS), Digital Span Forward Test (DSF), Digital Span Backward Test (DSB), Trail Making Test-A (TMT-A), Trail Making Test-A times (TMT-A times), Trail Making Test-A errors (TMT-A errors), or Repeatable Battery for the Assessment of Neuropsychological Status (RBANS); (d) memory measured by the Rivermead Behavior Memory Test (RMBT), Verbal learning test, Visual Learning Test, Forward Visual Span, or Backward Visual Span; (e) cognitive deterioration was measured by P300 latency or P300 amplitude. The secondary outcome indicators include (1) activities of daily living measured through the Barthel Index (BI), Modified Barthel Index (MBI), or Functional Independence Measure (FIM); (2) depression measured by the Beck Depression Inventory (BDI). Safety: adverse reactions.

### 2.3 Exclusion criteria

Six categories of literature were not considered in the study: (1) no definitive diagnosis of PSCI; (2) other types of literature were excluded, such as meta-analyses and systematic reviews on rTMS treatment for PSCI, which included animal experiments, cohort studies, case–control studies, and cross-sectional studies; (3) repeatedly published literature; (4) dissertations or conference articles; (5) MAs/SRs with less than five studies included; and (6) literature that compares two rTMS approaches.

### 2.4 Search strategy

Two authors independently searched PubMed, Cochrane Library, Embase, Web of Science, China Knowledge Network (CNKI), VIP database, WanFang database, and China Biomedical Literature Database (CBM) to collect MAs/SRs related to the efficacy of rTMS in improving PSCI. The search time limit was from the database construction to 26 March 2024. The search terms included stroke, cerebrovascular accident, transcranial magnetic stimulation, non-invasive brain stimulation, repetitive transcranial magnetic stimulation, cognitive dysfunctions, post-stroke cognitive impairment, systematic review, meta-analysis, etc. Also, the references of the included literature were traced to obtain the relevant MAs/SRs. The search strategy for PubMed is shown in Supplementary Table S1, and that for other databases is shown in Supplementary Table S2.

### 2.5 Literature screening and data extraction

Two authors (LZ and SG) independently searched the literature following the established search strategy. They performed quality assessment and initial screening based on title, abstract, and other relevant information. Subsequently, data extraction and verification were carried out to finalize the inclusion. In the event of disagreements, the third author (CW) reviewed the extracted information, and any inconsistencies were discussed among the three parties to reach a consensus. If the source material is incomplete, attempt to contact the author for additional information to enhance it. An evaluation information collection form was created using Excel 2016 software, encompassing publication year, design type, homogeneity, publication bias, AMSTAR-2 methodological quality score, and PRISMA report quality score.

### 2.6 Evaluation methods

#### 2.6.1 Methodological quality assessment

The AMSTAR-2 scale (25) is a revised version of the AMSTAR scale with 16 entries, 7 of which are vital entries (2, 4, 7, 9, 11, 13, and 15). Each entry is answered as “yes,” “partially yes,” or “no” based on the fulfillment of evaluation criteria. The AMSTAR-2 scale categorizes the methodological quality of MAs/SRs into four levels: “high” if ≤1 non-vital entry is defective; “moderate” if >1 non-vital entry is defective; “low” if one vital entry is defective with or without non-vital entries; and “very low” if >1 vital entry is defective with or without non-vital entries. Individual entries with a reporting rate ≤ 50% were considered to have missing reporting information, reporting rate = [(number of studies with full reporting of entries + number of studies with partial reporting)/total number of included studies] × 100%. Two authors (LZ and SG) independently evaluated the methodological quality of the included literature.

#### 2.6.2 Report quality assessment

The PRISMA Statement Inventory (26) assesses the quality of a study report based on the completeness of 27 entries. Each entry is scored as 1 for complete report, 0.5 for partial report, and 0 for failure to report, totaling 27 points. A score below 15 indicates a significant lack of information, 15 to 21 suggests some deficiencies, and > 21 to 27 indicates a relatively complete study. Two authors (LZ and SG) independently assessed the reporting quality of the literature included.

#### 2.6.3 Evidence quality assessment

The GRADE tool (27) assesses the quality of evidence for outcome indicators in MAs/SRs in five dimensions: publication bias, risk of bias, inconsistency, indirectness, and imprecision. The randomized controlled trials are graded as follows: high grade if there is no degradation in any of the five dimensions; moderate grade if there is a one-level degradation; low grade if there is a two-level degradation; and very low if there is a three-level degradation or more. Two researchers (LZ and SG) independently assessed the evidence quality of the included MAs/SRs. Any differences in their assessments were resolved through discussion with a third author (CW) to achieve a consensus.

#### 2.6.4 Radar plot evaluation entries included in the study

According to the method of Panesar et al. (28), a multidimensional analysis evaluation of the included MAs/SRs is conducted from six dimensions: qualitative evaluation of publication year, design type, homogeneity, and publication bias, as well as quantitative evaluation using the AMSTAR-2 methodological score and the PRISMA report quality score. In the AMSTRA-2 scale, a score of 1 is given for a “yes” evaluation, 0.5 for a “partially yes” evaluation, and 0 for a “no” evaluation (29). The PRISMA scale follows a scoring principle where 1 point is given for standardized and correct use of each entry, 0.5 points for inadequate use, and 0 points for incorrect use or failure to use, with a total of 27 points available (25). The more recent the publication year, the higher the evidence level of the literature. MAs/SRs were designated as high quality when the type of study included was an randomized controlled trial. When more than half of the included MAs/SRs have outcome indicators of *p* ≥ 0.01, I^2^ ≤ 50%, they will be judged as high homogeneity and assigned the highest rank, and if less than half, the literature will be judged as low homogeneity and assigned the lowest rank. When the literature uses a funnel plot or other methods for publication bias assessment, it will be judged as having low publication bias and assigned the highest rank; if not mentioned, it will be judged as having high publication bias and assigned the lowest rank. The ranking conversion of other projects is conducted according to the methods of medical statistics. The principle of ranking conversion is based on the methods of medical statistics. The highest ranking level is the total amount of literature, and the highest score for other indicators is assigned based on the total amount of literature. Finally, the evaluation rankings are included in the radar chart coordinates, and the average score of all rankings is taken as the ranking means.

#### 2.6.5 Radar plot drawing

The score sheet includes the basic information of the first author, publication year, design type, degree of homogeneity, and risk of publication bias evaluation scale. Also, it also records the scores of each methodological quality assessment of the AMSTAR-2 and the quality assessment of the PRISMA report. This information was imported into a table to draw and optimize a radar plot (30).

## 3 Results

### 3.1 Literature retrieval result

**Figure 1:**
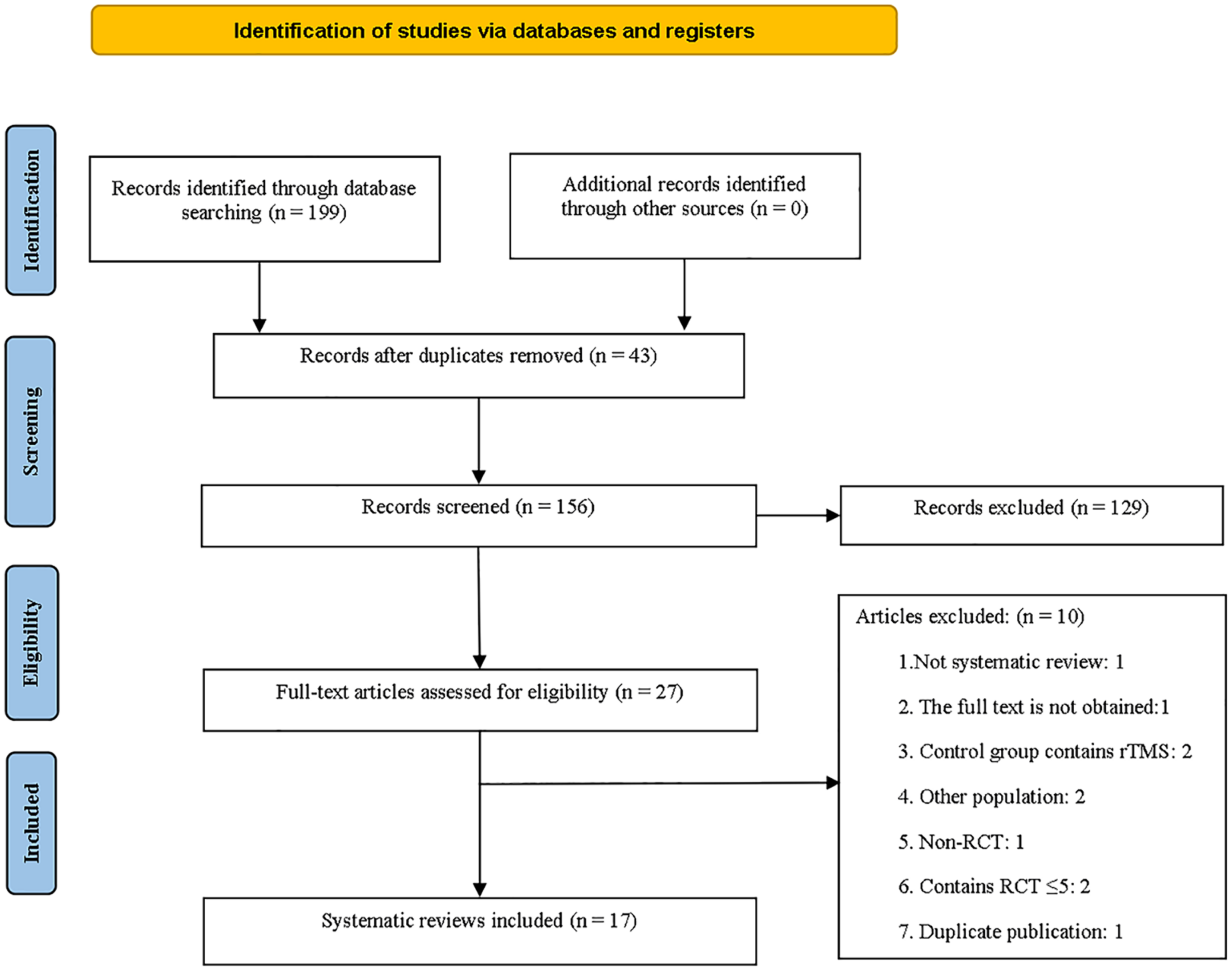
Flow chart of the literature search and study selection process.

A total of 199 documents were collected, and 156 documents remained after the removal of 43 duplicates by EndNote. Initially, 129 articles were excluded after reviewing their titles and abstracts. After conducting a more thorough analysis of the complete text, 10 articles were eliminated for different reasons. These reasons encompassed non-MAs/SRs, lack of complete texts, control groups incorporating rTMS, research involving patients with other neurological conditions, non-RCTs, MAs/SRs with less than five studies, and replicated published research. Finally, 17 studies met the criteria and were used for overviews (31–47). The specifics of the literature screening process are illustrated in Figure 1.

### 3.2 Basic features of the included literature

Seventeen articles (31–47) were included in the study, with 7 (31–35, 44, 45) in Chinese and 10 (36–43, 46, 47) in English, all published between 2021 and 2023. The MAs/SRs comprised only randomized controlled trials, with the studies varying from 5 to 61 and sample sizes ranging from 192 to 4,021 cases. All but one of the studies described the quality assessment tools used and meta-analyses of the results of the included studies. The risk of bias assessment tools used in most studies was the Cochrane and PEDro scales; one (41) study used the Modified Jadad scale scoring system and one (44) did not perform a risk of bias assessment. MMSE, MoCA, BI, MBI, RBMT, and LOTCA were among the primary outcome indicators used. Thirteen (31–41, 43, 47) articles reported financial support. Most studies concluded that rTMS has advantages in treating PSCI but emphasized the need for more, higher-quality studies to validate the results. For detailed information, refer to Table 1.

**Table 1:** Characteristics of included meta-analyses/systematic reviews.

Study ID	Country	Age (years)	Research type	Trials/n	Experimental group	Control group	Basic features of repetitive transcranial magnetic	Methodology evaluation tools	Outcomes	Main conclusion
Sun et al. (31)	China	No limit	Meta-analyses	9/767	rTMS + routine treatment +oxiracetam/butylphthalide/②/③; rTMS + ⑨; rTMS + ③ + ⑨/Donepezil Hydrochloride Capsules;	Routine treatment + ②/③/oxiracetam/butylphthalide; ⑨; ③ + ⑨/Donepezil Hydrochloride Capsules;	10 Hz, 4-12 weeks	Cochrane handbook for systematic reviews of interventions	Global cognition function: MoCA, MMSE; ADL: MBI	High-frequency repetitive transcranial magnetic stimulation can effectively improve the cognitive function of stroke patients and reduce the degree of neurological deficits. It also helps to improve the patients’ ability in daily life.
Yin et al. (32)	China	No limit	Meta-analyses	16/847	rTMS + ⑦/①/③; rTMS+⑦ + ① + ③; rTMS+① + psychotherapy; rTMS + ⑦ + psychotherapy; rTMS+⑦ + ①; rTMS	⑦ + ①/③; ①/③ + psychotherapy; ①/②/③/⑦; ① + ③;	1/10/5/Hz left DLPFC; 10 Hz DLPFC of lesion side; 1 Hz contralesional DLPFC; 3/5/10 Hz The left dorsolateral frontal lobe; 10 Hz Left prefrontal cortex; 3 Hz bilateral TL/FL/OL; 0.5 Hz bilateral FL	Cochrane Intervention Systematic Review Manual 5.1.0	Global cognition function: MMSE, MoCA; ADL: MBI; Cognitive deterioration: P300 latency, P300 amplitude	High-frequency and low-frequency repetitive transcranial magnetic stimulation therapy can effectively improve the cognitive function and daily living ability of stroke patients.
Liu et al. (33)	China	No limit	Meta-analyses	14/1,000	rTMS+⑦ + ① + ③; rTMS+⑦ + ①; rTMS+⑦; rTMS+⑧	Sham-rTMS + ⑦ + ① + ③; ⑦ + ① + ③; ⑦ + ①; ⑦/⑧	5 Hz DLPFC; 3/5/10 Hz left DLPFC; 10hz right DLPFC; 5/10 Hz DLPFC of lesion side; 3 Hz bilateral TL/FL/OL; 3 Hz not given	Cochrane handbook for systematic reviews of interventions	Global cognition function: MoCA; ADL: MBI; Cognitive deterioration: P300 latency, P300 amplitude	High-frequency rTMS can significantly improve cognitive function and activities of daily living in patients with PSCI.
Zhu et al. (34)	China	No limit	Meta-analyses	16/936	rTMS + ⑦/①; rTMS + ⑦ + ①; rTMS +⑦ + ① + Donepezil Hydrochloride Capsules/⑥/③; rTMS + ⑦ + ② + ③	⑦ + ①/③; sham-rTMS+⑦ + ①; sham-rTMS+⑦; ③/⑦; ① + Donepezil Hydrochloride Capsules	1–20 Hz, 3–8 weeks	Cochrane handbook for systematic reviews of interventions	Global cognition function: MoCA, MMSE; Other cognition performance: RBMT, LOTCA; ADL: MBI; Cognitive deterioration: P300 latency, P300 amplitude	TMS can significantly improve the cognitive function of patients after stroke, which is reflected in the improvement of cognitive scale score and the electrophysiological changes of TMS. The safety during treatment still needs special attention.
Wang and Han (35)	China	No limit	Systematic review	18/1, 235	rTMS + routine treatment; rTMS + routine treatment + Donepezil Hydrochloride Capsules; rTMS + ⑤; rTMS + Xueshuantong freeze-dried powder injection	⑤/routine treatment; routine treatment+Donepezil Hydrochloride Capsules; Xueshuantong freeze-dried powder injection; sham-rTMS + routine treatment	0.5–20 Hz, 20d–8 weeks	Cochrane handbook for systematic reviews of interventions	Global cognition function: MoCA; ADL: MBI; Cognitive deterioration: P300 latency, P300 amplitude	rTMS has shown promise in effectively improving cognitive function and enhancing the activities of daily living in individuals with PSCI.
Han et al. (36)	China	≥18	Systematic review, meta-analyses	19/847	rTMS/iTBS; rTMS/iTBS + routine treatment	Sham-rTMS/sham-iTBS; Placebo/blank control + routine treatment; Placebo/blank control	5/10/15/20 Hz left DLPFC; 10 Hz right DLPFC; 5/5–10/10/20 Hz DLPFC of lesion side	Cochrane handbook for systematic reviews of interventions, Physiotherapy evidence database scoring system	Global cognitive function: MMSE, MoCA; Other cognition performance: RMBT, TMT-A, DS, SCWT-C, SCWT-Cs; ADL: MBI, FIM; Cognitive deterioration: P300 latency, P300 amplitude; Depression: BDI	Stimulating the DLPFC region of the left hemisphere can improve the cognitive function of PSCI patients more than stimulating other parts, but it has no significant effect on improving ADL. In the future, more large-sample and high-quality studies are needed to explore.
Li et al. (37)	China	≥18	Systematic review, meta-analyses	10/347	rTMS + ①/⑦	Sham-rTMS + ①/⑦	5/10 Hz left DLPFC; 1 Hz right DLPFC; 1 Hz contralateral DLPFC; 5 Hz FDI cortical area	Cochrane handbook for systematic reviews Of interventions	Global cognitive function: MMSE, MoCA; Other cognition performance: RBMT, DST, DSF, DSB ADL: MBI; Depression: BDI	rTMS provides a non-invasive and effective technique for the treatment of post-stroke patients with cognitive impairment.
Xie et al. (38)	China	18–75	Systematic review, meta-analyses	5/192	rTMS; rTMS + ①/⑦	Sham-rTMS/no-rTMS/⑦/①; ① + ⑦	5 Hz left DLPFC	Cochrane risk-of-bias tool	Global cognition function: MoCA; Other cognition performance: RBMT; ADL: MBI	rTMS can improve stroke patients’ memory, global cognitive function, and ADL. Future research should focus on increasing follow-up time and the choice of placebo.
Chen et al. (39)	China	No limit	Systematic review, meta-analyses	34/2, 855	rTMS + routine treatment + ①/③/④/⑥/⑧; rTMS + routine treatment + ② + ③; rTMS + ①/②/③/⑨; rTMS + ① + ③ + ⑦; rTMS + ① + ③ + ⑦ + ②; rTMS + ③ + ⑦; rTMS + ③ + ⑦ + ②/⑧/⑤; rTMS + ① + ③/⑦; rTMS + ⑦ + ⑧	Routine treatment + ①/③/④/⑥; routine treatment + ① + ②/③; routine treatment +③ + ⑤; routine treatment + sham-rTMS + ①/③; routine treatment + sham-rTMS+③ + ②; sham-rTMS+①/③; sham-rTMS + ① + ⑦; sham-rTMS + ① + ③ + ⑦; sham-rTMS + ⑤ + ⑦; Sham-rTMS + ③ + ⑤ + ⑦; ①/②/⑨; ③ + ⑦; ③ + ⑦ + ①/②/⑧; ① + ③; ① + ② + ③ + ⑦	5/10-20 Hz DLPFC; 3/5/6/10/20 Hz, left DLPFC; 10 Hz right DLPFC; 10 Hz bilateral DLPFC; 5/10/5-10 Hz DLPFC of lesion side; 15 Hz bilateral TL, FL; 2 Hz bilateral TL, FL, OL; 31.25%MT-120%MT; 2–12 weeks	Cochrane Intervention Systematic Review Manual 5.1.0	ADL: BI, MBI, FIM	HF-rTMS can ameliorate ADL in patients with PSCI and has a better rehabilitation effect on PSCI.
Gao et al. (40)	China	>18	Systematic review, meta-analyses	8/36	rTMS + ①/⑤	Sham-rTMS + ①/⑤	1/5/10 Hz left DLPFC; 50 Hz burst repeated at 5 Hz (On/Off time2s/8 s) for 3 pulses, a total of 192 s and 600 pulses left DLPFC; 1 Hz right DLPFC; 1 Hz Contralateral DLPFC;	Physiotherapy evidence database scoring system	Global cognitive function: MMSE, MoCA Other cognition performance: Auditory CPT, visual CPT, Word of color word test, Color of color word test, Tower of London test, VST-C time, VST-C error words, LOTCA, DST, DSF, DSB, TMT-A times, TMT-A errors, RMBT, Verbal learning test, Visual learning test, Forward Visual span, Backward visual span; ADL: MBI	rTMS combined with cognitive training can improve the global cognitive function, executive function, working memory, and ADL of PSCI patients. However, there is a lack of evidence-level support, and further verification is needed in the future.
Xu et al. (41)	China	No limit	Systematic review, meta-analyses	10/591	rTMS + ①	Sham-rTMS + ①; ①	1–20 Hz; 4 weeks	Modified Jadad scale scoring system	Global cognition function: MoCA Other cognition performance: RBMT; ADL: MBI	rTMS might effectively improve the attention and memory impairment of stroke patients without increasing side effects. However, this effect needs to be verified by more multi-center, high-quality, large-sample, rigorously designed randomized controlled trials.
Liu et al. (42)	China	≥18	Systematic review, meta-analyses	6/197	rTMS/rTMS + other treatments	Sham-rTMS/⑦/①	1/10 Hz DLPFC; 10 Hz left DLPFC; 1 Hz right DLPFC; 0.5 Hz BFL; 1 Hz not given; 10–20 sessions	Cochrane Manual for Systematic Evaluation of Interventions	Global cognitive function: MMSE; Other cognition performance: LOTCA	rTMS has a positive effect on improving the cognitive ability of stroke patients. However, the evidence is still limited, and further large-scale studies are needed to explore the optimal stimulus parameters.
Gong et al. (43)	China	No limit	Systematic review, meta-analyses	12/497	rTMS/iTBS; iTBS + ①	Sham-rTMS /sham-iTBS/①	1 Hz contralateral DLPFC; 0.5 Hz bilateral DLPFC; 1/5/10 Hz left DLPFC; 50 Hz burst repeated at 5 Hz (On/Off time2s/8 s) for 3pulses, a total of 192 s and 600 pulses left DLPFC; 1 Hz right DLPFC; 1 Hz M1	Cochrane risk of bias tool	Global cognitive function: MMSE, MoCA; ADL: MBI; Other cognition performance: LOTCA	rTMS treatment on the DLPFC can improve cognitive function in patients with PSCI. There is no significant difference in the effect of high-frequency rTMS and low-frequency rTMS in patients with PSCl between high-frequency and low-frequency rTMS.
Li et al. (44)	China	No limit	Meta-analyses	6/240	rTMS+③; rTMS+③ + ⑦/①	③ + ⑦/①; ③/⑦/①	5/10 Hz affected/left hemisphere; 20-30 min; 2–4 weeks	None	Global cognitive function: MoCA	The high-frequency rTMS group was more effective than the conventional treatment group in treating post-stroke cognitive deficits, and high-frequency rTMS could promote the recovery of motor and cognitive functions.
Tian et al. (45)	China	No limit	Meta-analyses	17/1, 170	rTMS + ⑦ + ①/③; rTMS + ⑦/①/③; rTMS + ① + ③ + ⑧; rTMS + ⑦ + ① + ③	Sham-rTMS + ⑦ + ①/③; sham-rTMS + ⑦/①/③; sham-rTMS + ① + ③ + ⑧; sham-rTMS + ⑦ + ① + ③; ⑦ + ① + ③; ⑦/①/③; ⑦ + ①/③	1/5/10/15/5–10 Hz; 3 weeks—3 months	Cochrane risk of bias tool	Global cognitive function: MMSE, MoCA; ADL: MBI; Other cognition performance: LOTCA; Cognitive deterioration: P300 latency, P300 amplitude;	Repetitive transcranial magnetic stimulation can promote cognitive function recovery and improve ADL in PSCI patients with a good safety profile. Due to the limited number and quality of included studies, more high-quality studies are needed to verify the above conclusion.
Hara et al. (46)	Japan	18–85	Systematic review, meta-analyses	6/206	rTMS/iTBS + ③;	Sham-rTMS+ ③	1/10 Hz rTMS; L-DLPFC/R-DLPFC; iTBS:3 pulses of 50 Hz repeated at 5 Hz for a total of 190 s (600 pulses) 80/90/100% MT; 600–2,000 pulses/session; 10–20 sessions	Physiotherapy evidence database scoring system	Global cognitive function: MMSE, MoCA; ADL: FIM, BI; Other cognition performance: LOTCA, RBMT, TMT-A, DST, auditory-CPT, RBANS DS; Depression: BDI	The results demonstrate evidence of positive effects on cognitive functioning, including attention, WM, and memory. However, at present, there are only six studies for rTMS and 4 for tDCs, illustrating the need for further research. In terms of stimulation target and stimulation parameters, there is still limited evidence with significant variability.
Chen et al. (47)	China	No limit	Systematic review, meta-analyses	61/4, 012	rTMS; rTMS + routine treatment + ①/③/① + ③/③ + ⑤/Donepezil Hydrochloride Capsules; rTMS + ①/③/⑨/psychotherapy/Xueshuantong freeze-dried powder injection/Donepezil Hydrochloride Capsules; rTMS + ① + ③/① + ⑦/② + ⑦/③ + ⑦/⑤ + ⑦; rTMS + ③ + ⑦ + ①/②/⑤/⑧; rTMS + ① + ④ + ⑦ + ⑧	Sham-rTMS; sham-rTMS + routine treatment + ①/③/① + ③/③ + ⑤/Donepezil Hydrochloride Capsules; sham-rTMS + ①/③/⑨/psychotherapy/Xueshuantong freeze-dried powder injection/Donepezil Hydrochloride Capsules; sham-rTMS + ① + ③/① + ⑦/② + ⑦/③ + ⑦/⑤ + ⑦; sham-rTMS++ ③ + ⑦ + ①/②/⑤/⑧; sham-rTMS + ① + ④ + ⑦ + ⑧	2/3/5/6/10/15/20/5–10/10–20 Hz; 80–120% MT/3 mT; DLPFC/left-DLPFC/right-DLPFC/bilateral DLPFC/bilateral TL, FL, OL/M1 of lesion side/DLPFC of lesion side/PFC; 20 min × 2–3d/5d/6d/7d	Cochrane Intervention Systematic Review Manual 5.1.0.	Global cognitive function: MMSE, MoCA, ADAS-Cog; Other cognition performance: LOTCA; Cognitive deterioration: P300 latency, P300 amplitude;	Compared to non-rTMS or sham rTMS, HF-rTMS not only improves the global cognitive function of PSCI patients but also has better rehabilitation results.

① cognition training; ② acupuncture; ③ rehabilitation therapy; ④ oxiracetam; ⑤ computer-assisted cognitive rehabilitation; ⑥ wuling capsules; ⑦ pharmacotherapy; ⑧ hyperbaric oxygen; ⑨ occupational therapy; TL, temporal lobe; FL, frontal lobe; OL, occipital lobe; M1, primary motor cortex M1 area; BFL, bilateral frontal lobe; FDI, first dorsal interosseous; DLPFC, dorsolateral prefrontal cortex; MT, motor threshold; mT, milliteslas; HF-rTMS, High frequency rTMS; None, means not stated or not used in the text.

### 3.3 Comparison of the publication year

The significance of each research study on clinical guidance is related to the publication year, scope, and duration. Newer studies and those covering longer time spans contribute to more vital guidance for clinical practices. This study included 17 MAs/SRs. Therefore, the literature rankings published in 2023 were positioned as 17. The earliest publication date is 2021, containing five (31, 33, 34, 42, 46) studies; the latest is 2023, containing 10 (32, 36–40, 43–45, 47). Meanwhile, there were two (35, 41) studies in 2022.

### 3.4 Comparison of study design

The most compelling form of clinical evidence for assessing the efficacy of a treatment is an randomized controlled trial.

### 3.5 Results of the methodological quality assessment

The methodological quality of the included 17 (31–47) articles was evaluated by AMSTAR-2, and the results showed that the methodological quality of the included 17 MAs/SRs was deficient. The vital entries reported as missing in large numbers are entry 2 (41%), entry 7 (0%), entry 13 (12%), and entry 15 (41%). The following were the primary causes: (1) only seven (36–38, 40, 43, 46, 47) articles were numbered and registered in the program before the evaluation; (2) the reasons for the exclusion list are not provided in all the articles; (3) only two (40, 46) articles considered the risk of study bias when discussing or analyzing the results of each study; (4) all articles were searched without consulting experts in the relevant fields; and (5) 10 (31, 33, 36, 39–44, 47) articles do not describe the likelihood of the existence of publication bias in the small-sample studies and the severity of their impacts. The non-vital entries reported as missing in large numbers are entry 3 (0%), entry 10 (0%), entry 12 (29%), and entry 14 (47%). The main reasons were as follows: (1) none of the studies explained the reasons for the inclusion of RCT; (2) all of the literature did not describe the essential characteristics of the included studies in detail; (3) only six (36–38, 41, 46, 47) articles assessed the risk of bias of the included studies on the potential impact on the study results; (4) The authors of the nine (33, 35, 37, 41–46) studies did not provide a satisfactory explanation or discussion of the heterogeneity in the results of the MAs/SRs (Supplementary Table S3). Seventeen articles had a score ranging from 5 to 11. Among them, three (33, 42, 44) studies scored 5, one (31) literature scored 6, one (41) literature scored 6.5, four (35, 39, 43, 45) articles scored 7, one (34) literature scored 8, one (37) literature scored 9, three (32, 36, 40) articles scored 10, and three (38, 46, 47) articles scored 11. Refer to Table 2 for details.

**Table 2:** Multivariate evaluation of the six dimensions and rank of the included literature.

Study ID	Publication year	Design type	AMSTAR-2 scale	PRISMA scale	Publication bias	Homogeneity	Average rank score
Sun et al. (31)	2021 (5)	RCT (17)	6 (4)	18 (9)	None (10)	Low (9)	9.00
Yin et al. (32)	2023 (17)	RCT (17)	10 (14)	22 (16)	Bgger’ s and Egger’ s tests (17)	High (17)	16.33
Liu et al. (33)	2021 (5)	RCT (17)	5 (3)	12 (1)	None (10)	High (17)	8.83
Zhu et al. (34)	2021 (5)	RCT (17)	8 (10)	17 (7)	Funnel plot (17)	Low (9)	10.83
Wang and Han (35)	2022 (7)	RCT (17)	7 (9)	16.5 (3)	Funnel plot (17)	High (17)	11.67
Han et al. (36)	2023 (17)	RCT (17)	10 (14)	20.5 (13)	None (10)	Low (9)	13.33
Li et al. (37)	2023 (17)	RCT (17)	9 (11)	20 (12)	Funnel plot (17)	High (17)	15.17
Xie et al. (38)	2023 (17)	RCT (17)	11 (17)	25.5 (17)	Bgger’ s and Egger’ s tests (17)	High (17)	17.00
Chen et al. (39)	2023 (17)	RCT (17)	7 (9)	20 (12)	None (10)	High (17)	13.67
Gao et al. (40)	2023 (17)	RCT (17)	10 (14)	21 (14)	None (10)	High (17)	14.83
Xu et al. (41)	2022 (7)	RCT (17)	6.5 (5)	17 (7)	Funnel plot (17)	Low (9)	10.33
Liu et al. (42)	2021 (5)	RCT (17)	5 (3)	17 (7)	None (10)	Low (9)	8.50
Gong et al. (43)	2023 (17)	RCT (17)	7 (9)	20 (12)	None (10)	High (17)	13.67
Li et al. (44)	2023 (17)	RCT (17)	5 (3)	16.5 (3)	None (10)	Low (9)	9.83
Tian et al. (45)	2023 (17)	RCT (17)	7 (9)	22 (16)	Funnel plot (17)	Low (9)	14.17
Hara et al. (46)	2021 (5)	RCT (17)	11 (17)	17 (7)	None (10)	Low (9)	10.83
Chen et al. (47)	2023 (17)	RCT (17)	11 (17)	18 (9)	None (10)	Low (9)	13.17
Average score of evaluation entries	12.29	17	9.88	9.71	12.88	12.76	12.42

“None” means not stated or not used in the text; numbers in parentheses represent the rank order of each document in the total literature.

### 3.6 Results of the quality assessment of literature reports

Every PRISMA statement entry with a reporting completeness of less than 50% was deemed to be considerably underreported. Entry 7 (retrieval; 35%), entry 10 (information; 50%), entry 14 (study bias; 47%), entry 15 (outcome evidence quality; 41%), entry 22 (presentation of the quality of outcome evidence; 41%), entry 24 (other information; 21%), and entry 27 (shared information; 18%) were the entries with the most significant information deficiencies. The remaining 20 entries reported a good level of completeness (Supplementary Table S4). According to the PRISMA scale, 17 articles scored between 12 and 25.5. No perfect scores were recorded, with only one (33) article scoring below 15. Thirteen (31, 34–37, 39–44, 46, 47) articles reported scores from 15 to 21, and four (32, 38, 45) articles reported scores from 21 to 27. Refer to Table 2 for details.

### 3.7 Radar plot for multi-dimensional evaluation items

Refer to Table 2 for details.

### 3.8 Results of radar plot evaluation

**Figure 2:**
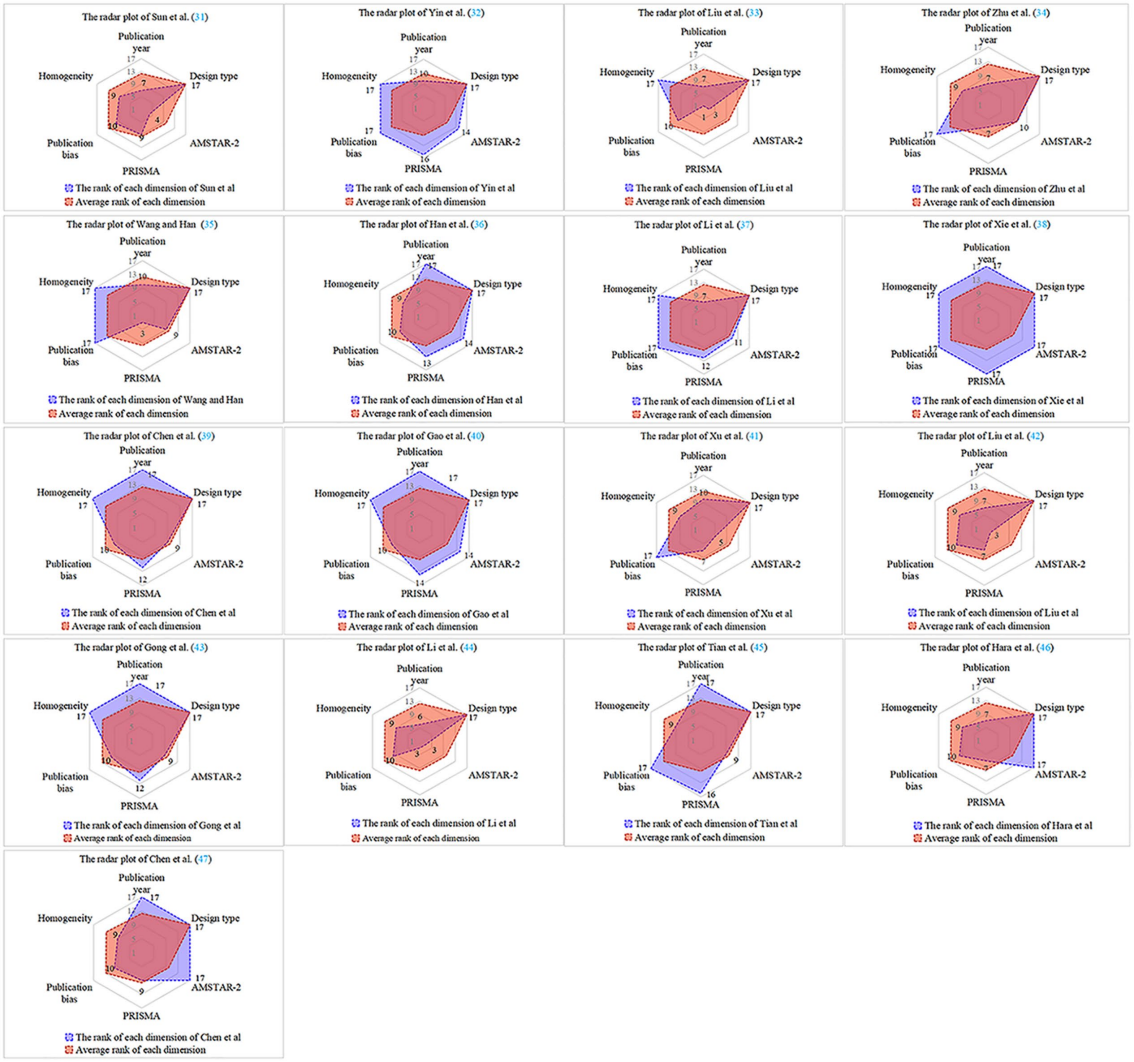
Radar plot for inclusion literature.

The radar plot demonstrates that all six evaluation dimensions of Xie et al. (38) are higher than the rank average score, with a larger radar plot area and the highest significance in literature references. Three studies (33, 41, 46) had four evaluation dimensions below the rank average score, with smaller radar plot areas and lesser literature reference significance. Three studies (31, 42, 44) had five evaluation dimensions below the rank average score, with the smallest radar plot areas, and references to this type of literature must be considered with caution. Overall, the quality of literature published during 2021–2023 is variable, and its methodological and reporting quality needs to be improved. Refer to Figure 2 for details.

### 3.9 Results of the GRADE evidence quality rating evaluation

The quality of evidence was evaluated for eight outcome indicators and 131 pieces of evidence related to global cognitive functioning, executive functioning, working memory, memory, attention, ADL, depression, and cognitive deterioration in the 17 articles included in the study. The GRADE approach was utilized to evaluate and analyze the strength of evidence for these outcome indicators. Evidence-based RCT was initially classified as high quality, but this classification could change due to publication bias, risk of bias, inconsistency, imprecision, and indirectness (48). Ultimately, the GRADE quality assessment revealed that 51 evidence were of very low quality, 67 were of low quality, one was of high quality, and 12 were of moderate quality (Supplementary Table S5).

## 4 Adverse events

Thirteen studies included in the analysis (31–34, 36, 38–41, 43, 45–47) mentioned the occurrence of adverse effects following rTMS therapy. The frequently mentioned adverse effects were mild headache, dizziness, nausea, and vomiting, as well as other effects, including loss of appetite, anxiety, and fatigue. However, these symptoms reportedly lessened after a brief period of rest. This suggests that rTMS is a safe and effective therapeutic method for managing post-stroke cognitive impairment in patients.

## 5 Discussion

### 5.1 Summary of evidence

This study included 17 (31–47) MAs/SRs based on RCTs published between 2021 and 2023. Seven were in Chinese (31–35, 45, 46), and 10 were in English (36–43, 46, 47). The average rank score of the 17 included articles ranged from 8.50 to 17.00, with an overall average rank score of 12.42. Xie et al. (38) had the highest quality articles with an average rank score of 17.00, while Liu et al. (42) had the lowest quality articles with an average rank score of 8.50. Only two articles (33, 44) did not apply a forest plot to the stated results. Seventeen articles had AMSTRA-2 scale scores of 5–11 and PRISMA scale scores of 12–25.5. Eight articles (31, 32, 34, 36, 38–40, 47) have addressed and discussed the heterogeneity and documented the high levels of heterogeneity. The low scores were primarily caused by low methodological quality and report quality. Those that need to be strengthened in terms of methodological quality are: (1) the search must be thorough and methodical, considering the language of publication and conducting a thorough search of gray literature to avoid query bias; (2) when documenting the included studies, it is essential to provide comprehensive details on the subjects, interventions, outcome indicators, study design, and study location; (3) the risk of bias in included studies should be assessed using appropriate evaluation tools, emphasizing the rational use of funnel plots or other tools to report publication bias; and (4) research funding sources and related support descriptions. Those that need to be strengthened in terms of quality of reporting are: (1) the introduction should describe the research on clinical issues according to the PICOS principle; (2) in order to ensure transparency in research, studies need to be registered in advance in an international preregistration database, outlining the research plan; (3) the researcher should rationally explain and discuss the heterogeneity of the results in the report of the findings; and (4) in the discussion section, in addition to explaining the research findings of the article, it is also necessary to extensively discuss the limitations of its inclusion in the article. The above illustrates the low quality of MAs/SRs regarding rTMS treatment of PSCI patients, and there is still much room for improvement.

### 5.2 Pathogenesis of post-stroke cognitive impairment

Post-stroke cognitive impairment, a common complication following a stroke, is characterized by attention deficit and memory impairment. These symptoms significantly affect the capacity of rehabilitation and adapt to society. On one hand, post-stroke motor and cognitive deficits impact the implementation of rehabilitation programs by stroke patients. On the other hand, PSCI reduces patients’ motivation and initiative for rehabilitation therapy, affecting its efficacy and slowing down the rehabilitation process, which in turn has a significant influence on the family and society. There is an urgent need to create safer, more effective, and more acceptable rehabilitation treatments because the biomedical-medical-psychological paradigm has changed, and traditional rehabilitation treatments are highly limited in enhancing the patient’s level of functioning.

The process of PSCI pathologizing is still not fully understood. After stroke, ischemia leads to the emergence of an “ischemic penumbra” at the site of the lesion and in the surrounding area, and the expression of apoptosis-related proteins increases with the duration and degree of ischemia, resulting in cell death and neurological damage (49). As early as 1970, Tomlinson et al. (50) suggested that the development of cognitive dysfunction is linked to the volume of cerebral infarction. If the volume exceeds 100 mL, it may result in vascular dementia. Additionally, Zekry et al. (51) discovered that infarctions in specific brain regions, such as the frontal lobe and white matter, have a more pronounced impact on cognitive deficits, highlighting the crucial role of infarct location in the mechanism of cognitive impairments. They also concluded that the volume of cerebral infarcts only accounted for some of the cognitive decline in stroke patients and that the number of foci of infarcts was significantly correlated with the severity of cognitive deficits. PSCI was previously thought to be connected to damage in specific brain regions like the internal olfactory cortex and the hippocampus, which are associated with Alzheimer’s disease (AD) (52). Individuals with AD and PSCI experience similar neuropathological processes, and research has revealed that individuals with PSCI exhibit AD-specific abnormalities, such as elevated amyloid beta-protein deposition (53). Furthermore, research has shown that cognitive impairment after a stroke is linked not only to the localization of the lesion but also to the distal areas of the lesion and the broader neural networks. For instance, there is a correlation with decreased functional connectivity within the default mode networks of the medial temporal lobe, posterior cingulate gyrus, and medial prefrontal cortex (54). Moreover, the pathological processes leading to PSCI may involve reduced blood flow in the brain, an inflammatory response, damage from oxygen radicals, and neurodegeneration of the brain (55–57). However, engaging in activities of daily living requires good motor skills and cognitive resource participation, which may involve analyzing, understanding, and learning daily activities. Enhancing and restoring cognitive function is as important as recovering motor function for patients to reintegrate fully into their families and society.

### 5.3 Possible mechanisms of repetitive transcranial magnetic stimulation for cognitive impairment

Cognitive function plays a critical role in predicting the progression of a stroke and is an essential determinant of the patient’s post-stroke quality of life. Addressing cognitive impairment through effective treatment is of utmost importance. Here are several potential mechanisms through which rTMS, a widely used non-invasive treatment for enhancing cognitive function, operates: (1) rTMS is a significant tool for reconstructing part or all of the functional neural network. It not only brings about changes in the local cortical areas but also alters the function of the connected remote subcortical areas. Additionally, the improvement in function persists even after the stimulation is ceased (58). The neuroprotective effects of rTMS against cerebral ischemia may be achieved through enhancing blood oxygenation and local cerebral blood flow velocity, stimulating the expression of brain-derived neurotrophic factor (BDNF) in the brain tissue surrounding the infarct, and reducing ischemic injury-induced neuronal apoptosis in the peri-ischemic brain area. Moreover, BDNF enhances learning and memory by promoting synaptic transmission and neuronal plasticity in the central nervous system (59). Research has demonstrated that neurological disorders are linked to abnormal levels of brain metabolites, and it has been demonstrated that rTMS can normalize these levels and improve cognitive function (60). Resting-state functional magnetic resonance imaging (RS-fMRI) is an effective method for investigating structural and functional changes in the brain. In recent years, much RS-fMRI research has focused on neuroplasticity and treatment response related to rTMS. Maintaining optimal interactions between brain networks to enhance cognitive performance is crucial, and rTMS plays a role in enhancing functional connections within these networks to improve cognitive function (61). (2) Research suggests that the mechanism by which rTMS enhances cognitive function may be through the modulation of antioxidant enzymes (62). rTMS can regulate neurotransmitters, including dopamine and gamma-aminobutyric acid (GABA). For example, rTMS on the dorsolateral prefrontal cortex (DLPFC) increases extracellular dopamine levels and regulates dopamine release in the anterior cingulate cortex and orbitofrontal cortex, where dopamine plays a vital role in cognitive processes. (3) rTMS not only affects the stimulated area but also induces changes in cortical and subcortical functional connectivity. The prefrontal-striatal neural circuit is an important cognitive control center that plays a dominant role in behavioral inhibition, impulse control, and decision-making, and increasing its connectivity improves cognitive function (63). The study found that after TMS was applied to the DLPFC of healthy subjects, it was found to induce neuronal activity in several connected brain regions by functional magnetic resonance imaging (fMRI). Furthermore, it was found that this area and other prefrontal cortex regions are involved in attention and memory processes (64). The hippocampus is a part of the brain mainly involved in learning and memory. The granule cells of the hippocampal dentate gyrus are involved in spatial navigation processes based on location strategies, participating in the temporary storage of spatial information, and are closely related to anxiety control and contextual learning. Research on rats has shown that rTMS enhances the excitability of hippocampal dentate gyrus granule cell neurons, playing a positive role in promoting cognitive function recovery (65). When cognitive impairments occur, various resting-state networks of patients (such as attention and sensory-motor networks) all undergo abnormalities. These networks interact with each other and collectively control attention and other cognitive domains. After receiving rTMS treatment, it was discovered through fMRI that rTMS promotes normalizing abnormal resting-state network structures (66). (4) rTMS can alter the structure of dendritic spines and the expression of synaptic proteins, neurotransmitters, and cognition-related metabolites. rTMS can also modulate synaptic transmission, resulting in long-term potentiation or inhibition associated with learning and memory (67). (5) Studies have shown that rTMS can regulate the levels of growth factors. Vascular endothelial growth factor (VEGF) has been identified as a neurotrophic factor in the peripheral nervous system, which can improve cell survival and promote cell proliferation and axon growth. VEGF in the hippocampus area can enhance learning and memory. Insulin-like growth factor is an influential growth factor in the central nervous system, which can significantly reduce brain damage caused by hypoxia and improve long-term memory and cognitive abilities in rats. Erythropoietin is expressed in the cortex and hippocampal neurons and is associated with high synaptic plasticity and cognitive function (68). rTMS-induced increase in amyloid precursor protein levels may mediate neuroprotective effects, preventing oxidative death of nerve cells and thereby improving cognitive function (69).

### 5.4 Current status of research on repetitive transcranial magnetic stimulation for the treatment of post-stroke cognitive impairment

This study summarizes the features of current clinical rTMS applications for treating PSCI patients. (1) These include a standard stimulation frequency of 1 Hz for low-frequency stimulation and 10 Hz for high-frequency stimulation. Three studies (38, 41, 43) comparing the effectiveness of high and low-frequency stimulation concluded that the two had no significant difference in improving cognitive function. (2) The DLPFC, including the left, right, contralateral, and affected DLPFC, is a commonly targeted site for stimulation. Among these, the left DLPFC is the most frequently stimulated. It is important to note that the stimulation frequency varies across studies for the same stimulation site. High-frequency stimulation of the DLPFC is a common approach, but its actual effects still need to be determined. (3) No specific guidelines recommend stimulation intensity, but 80% to 120% is typically utilized in clinical treatment. (4) The intervention time and period varied across the studies. The most common session duration was 20 min, with the most extended period lasting 12 weeks and the shortest lasting 1 week. The majority of studies had an intervention period of 4 weeks. Forty sessions of low-frequency stimulation were more effective than 20 or 10 sessions, and 15 sessions of high-frequency stimulation were more productive than 20 or 10 sessions. (5) The effect of stimulation during the acute or subacute phase of stroke is more significant than during the chronic phase, according to the post-stroke duration study (40). (6) The details of the rTMS treatment regimen, such as the number of sessions, the time between sessions, and the number of pulses in each session, are not well-documented. (7) The predominant adverse effects reported in the MAs/SRs examined in this research were headache and dizziness. These symptoms were alleviated by rest, suggesting that rTMS could have broad utility in real-world scenarios.

There are several limitations to using rTMS in treating PSCI clinical trials. (1) The effectiveness of rTMS for PSCI and the degree of its effectiveness remain uncertain, as most of the RCT experimental groups were administered rTMS alongside other treatments. (2) The excitation thresholds of different areas in the cortex vary. Most recent studies have focused on measuring the motor thresholds of the patient’s motor cortex to gauge the stimulus intensity. However, it remains unclear whether the stimulus intensity derived from motor cortex stimulation can be extrapolated to the stimulation of cognitive functional areas. (3) Much of the research has emphasized the activation of the DLPFC, but other functional regions, including the primary motor cortex, temporal lobe, and frontal lobe, have also been explored. The findings indicate that rTMS may enhance cognitive function in PSCI patients. However, it remains unclear whether stimulating multiple sites yields more pronounced effects than stimulating a single site. (4) Repetitive transcranial magnetic stimulation is a rather complex non-pharmacological intervention method. In clinical applications, it is often used as an adjuvant therapy. Determining relevant stimulation parameters, such as stimulation site, frequency, intensity, and duration, mainly relies on the therapist’s experience or references to previous studies without considering individual differences. Future clinical research should consider the individual characteristics of PSCI patients to improve the reliability, accuracy, and replicability of trials.

The research has a few limitations: (1) Only Chinese and English articles were included, and paper or gray articles were not searched, so there may have been omissions. (2) The study only included 17 pieces of literature, with an average quality rank score of 12.42. The GRADE quality of evidence score was mainly low-quality, and the limited number and low quality of the literature may impact the scientific validity and accuracy of the study results. (3) Despite cross-assessment by our study members, the results of this study are inevitably somewhat subjective. Therefore, clinical staff should consider the actual situation when using this data for clinical decision-making.

## 6 Conclusion

In conclusion, this study demonstrates the need to further improve the quality of the literature on MAs/SRs of transcranial magnetic stimulation for treating patients with post-stroke cognitive impairment. To ensure higher-quality evidence for clinical use, researchers conducting MAs/SRs should rigorously adhere to the AMSTAR-2 and PRISMA standards, enhancing the quality of analytical approaches and reports. Although current evidence suggests that rTMS enhances global cognitive function and activities of daily living in PSCI patients, this should be corroborated in future studies, considering issues such as low-quality and limited articles.

## Data availability statement

The original contributions presented in the study are included in the article/Supplementary material, further inquiries can be directed to the corresponding authors.

## Author contributions

LZ: Conceptualization, Formal analysis, Methodology, Supervision, Writing – original draft. SG: Conceptualization, Formal analysis, Methodology, Supervision, Writing – original draft. CW: Data curation, Formal analysis, Methodology, Writing – review & editing. YL: Data curation, Formal analysis, Methodology, Writing – review & editing. HY: Data curation, Formal analysis, Methodology, Writing – review & editing. LC: Conceptualization, Data curation, Funding acquisition, Supervision, Validation, Writing – review & editing. CG: Funding acquisition, Writing – review & editing.

## References

[1] Zhang X Bi X. Post-stroke cognitive impairment: a review focusing on molecular biomarkers. J Mol Neurosci. (2020) 70:1244–54. doi: 10.1007/s12031-020-01533-8, PMID: 32219663

[2] Wang Y Xu N Wang R Zai W. Systematic review and network meta-analysis of effects of non-invasive brain stimulation on post-stroke cognitive impairment. Front Neurosci. (2022) 16:1082383. doi: 10.3389/fnins.2022.1082383, PMID: 36643019 PMC9832390

[3] Snyder HM Corriveau RA Craft S Faber JE Greenberg SM Knopman D . Vascular contributions to cognitive impairment and dementia including Alzheimer’s disease. Alzheimers Dement. (2015) 11:710–7. doi: 10.1016/j.jalz.2014.10.008, PMID: 25510382 PMC4731036

[4] Lin YF Wei TY Zhang XY Li CJZ He JJ Du XX. Effect of music therapy on post-stroke cognitive impairment. Chin J Rehabil Theory Pract. (2023) 29:714–9. doi: 10.3969/j.issn.1006⁃9771.2023.06.013

[5] Mijajlović MD Pavlović A Brainin M Heiss W-D Quinn TJ Ihle-Hansen HB . Post-stroke dementia – a comprehensive review. BMC Med. (2017) 15:11. doi: 10.1186/s12916-017-0779-7, PMID: 28095900 PMC5241961

[6] Lei XX Song LP. Effect of cognitive training based on PASS theory on post-stroke cognitive impairment. Chin J Rehabil Theory Pract. (2020) 26:70–6. doi: 10.3969/j.issn.1006⁃9771.2020.01.013

[7] Chinese Society of Geriatrics Hypertension Branch, Beijing hypertension prevention and control association, National Clinical Medical Research Center for geriatric diseases (Xuanwu Hospital of Capital Medical University, general Hospital of the Chinese People’s liberation Army) . Guidelines for the management of hypertension in the elderly in China 2023. Chin J Hypertens. (2023) 31:508–38. doi: 10.16439/j.issn.1673-7245.2023.06.003

[8] Hu YX Guo YF Wang L. Chinese expert consensus on cognitive impairment in hypertension in the elderly. Chin J Clin Healthc. (2021) 24:145–59. doi: 10.3969/J.issn.1672-6790.2021.02.001

[9] Ruan XD Gao J Lü Z Li Q Su KQ Gu YM . Effect of Tongdu Xingshen acupuncture on the expression of AMPA receptor and its auxiliary protein in hippocampus of rats with learning and memory impairment after cerebral ischemia reperfusion. J Tradit Chin Med. (2023) 64:2435–42. doi: 10.13288/j.11-2166/r.2023.23.011

[10] Sun QQ Xie F Yao L Lü ZM Zhong ML. Research progress of Chinese medicine treatment for mild cognitive impairment. J Shaanxi Univ Chin Med. (2023) 46:127–30. doi: 10.13424/j.cnki.jsctcm.2023.03.024

[11] Traut HJ Guild RM Munakata Y. Why does cognitive training yield inconsistent benefits? A meta-analysis of individual differences in baseline cognitive abilities and training outcomes. Front Psychol. (2021) 12:662139. doi: 10.3389/fpsyg.2021.662139, PMID: 34122249 PMC8187947

[12] Chen LJ Li J Chu J. Rehabilitation robots in stroke patients. Shanghai Nurs. (2023) 23:72–5. doi: 10.3969/j.issn.1009-8399.2023.03.016

[13] Zhu S Sui Y Shen Y Zhu Y Ali N Guo C . Effects of virtual reality intervention on cognition and motor function in older adults with mild cognitive impairment or dementia: a systematic review and meta-analysis. Front Aging Neurosci. (2021) 13:586999. doi: 10.3389/fnagi.2021.586999, PMID: 34025384 PMC8136286

[14] Li Y Luo H Yu Q Yin L Li K Li Y . Cerebral functional manipulation of repetitive transcranial magnetic stimulation in cognitive impairment patients after stroke: An fMRI study. Front Neurol. (2020) 11:977. doi: 10.3389/fneur.2020.00977, PMID: 33013646 PMC7506052

[15] Yin M Liu Y Zhang L Zheng H Peng L Ai Y . Effects of rTMS treatment on cognitive impairment and resting-state brain activity in stroke patients: a randomized clinical trial. Front Neural Circuits. (2020) 14:563777. doi: 10.3389/fncir.2020.563777, PMID: 33117131 PMC7561423

[16] Ganho-Ávila A Poleszczyk A Mohamed MMA Osório A. Efficacy of rTMS in decreasing postnatal depression symptoms: a systematic review. Psychiatry Res. (2019) 279:315–22. doi: 10.1016/j.psychres.2019.05.042, PMID: 31196691

[17] O'Brien AT Bertolucci F Torrealba-Acosta G Huerta R Fregni F Thibaut A. Non-invasive brain stimulation for fine motor improvement after stroke: a meta-analysis. Eur J Neurol. (2018) 25:1017–26. doi: 10.1111/ene.13643, PMID: 29744999

[18] Fisicaro F Lanza G Grasso AA Pennisi G Bella R Paulus W . Repetitive transcranial magnetic stimulation in stroke rehabilitation: review of the current evidence and pitfalls. Ther Adv Neurol Disord. (2019) 12:175628641987831. doi: 10.1177/1756286419878317, PMID: 31598137 PMC6763938

[19] Yingli B Zunke G Wei C Shiyan W. Cerebral activity manipulation of low-frequency repetitive transcranial magnetic stimulation in post-stroke patients with cognitive impairment. Front Neurol. (2022) 13:951209. doi: 10.3389/fneur.2022.951209, PMID: 36425802 PMC9679635

[20] Gillespie DC Bowen A Chung CS Cockburn J Knapp P Pollock A. Rehabilitation for post-stroke cognitive impairment: an overview of recommendations arising from systematic reviews of current evidence. Clin Rehabil. (2015) 29:120–8. doi: 10.1177/0269215514538982, PMID: 24942480

[21] Lorenz RC Matthias K Pieper D Wegewitz U Morche J Nocon M . A psychometric study found AMSTAR 2 to be a valid and moderately reliable appraisal tool. J Clin Epidemiol. (2019) 114:133–40. doi: 10.1016/j.jclinepi.2019.05.028, PMID: 31152864

[22] Sarkis-Onofre R Catalá-López F Aromataris E Lockwood C. How to properly use the PRISMA statement. Syst Rev. (2021) 10:117. doi: 10.1186/s13643-021-01671-z, PMID: 33875004 PMC8056687

[23] Pollock M Fernandes RM Pieper D Tricco AC Gates M Gates A . Preferred reporting items for overviews of reviews (PRIOR): a protocol for development of a reporting guideline for overviews of reviews of healthcare interventions. Syst Rev. (2019) 8:335. doi: 10.1186/s13643-019-1252-9, PMID: 31870434 PMC6929355

[24] Page MJ McKenzie JE Bossuyt PM Boutron I Hoffmann TC Mulrow CD . The PRISMA 2020 statement: An updated guideline for reporting systematic reviews. Int J Surg. (2021) 88:105906. doi: 10.1016/j.ijsu.2021.105906, PMID: 33789826

[25] Zhang FY Shen AM Zeng XT Qiang WM Jin YH. An introduction to AMSTAR 2: a critical appraisal tool for systematic reviews. Chin J Evid Based Cardiovasc Med. (2018) 10:14–8. doi: 10.3969/j.issn.1674-4055.2018.01.03

[26] Page MJ McKenzie JE Bossuyt PM Boutron I Hoffmann TC Mulrow CD . Statement: an updated guideline for reporting systematic reviews. BMJ. (2020) 372:n71. doi: 10.1136/bmj.n71, PMID: 33782057 PMC8005924

[27] Goldet G Howick J. Understanding GRADE: an introduction. J Evid Based Med. (2013) 6:50–4. doi: 10.1111/jebm.12018 23557528

[28] Panesar SS . Development of the veritas plot and its application in cardiac surgery: an evidence-synthesis graphic tool for the clinician to assess multiple meta-analyses reporting on a common outcome. Can J Surg. (2009) 52:E137–45. 19865543 PMC2769126

[29] Hu NN Guo H Lin KK Zhang A Chen SS. Effects of aquatic therapeutic exercise in stroke rehabilitation: an overview of systematic reviews. Chin Gen Pract. (2022) 25:2421–8. doi: 10.12114/j.issn.1007-9572.2022.0249

[30] Wang XT Lin HX Chen GZ. Systematic reviews /meta analysis of acupuncture therapy on post-stroke depression based on multiple evaluation of radar plot. Chin J Basic Med Tradit Chin Med. (2018) 24:518–22. doi: 10.19945/j.cnki.issn.1006-3250.2018.04.034

[31] Sun CJ Zhan TT Wang Y Wang LL Li X. A systematic evaluation of the effect of high frequency repeated transcranial magnetic stimulation on cognitive function of stroke. J MuDanJiang Med Univ. (2021) 42:51–55+134. doi: 10.13799/j.cnki.mdjyxyxb.2021.05.014

[32] Yin YK Wang JL Sun JZ. Therapeutic effect of different-frequency repetitie transcranial magnetic stimulations on post-stroke cognitive impairment: a Meta-analysis. Chin J Tissue Eng Res. (2023) 27:3274–80. doi: 10.12307/2023.150

[33] Liu W Hu P Huo MX Feng B. The efficacy of high-frequency repetitive transcranial magnetic stimulation in the treatment of post-stroke cognitive dysfunction Meta-analysis. Chin J Phys Med Rehabil. (2021) 43:1021–5. doi: 10.3760/cma.j.issn.0254-1424.2021.11.015

[34] Zhu MY Lu Y Dai XY Liu YB. Clinical efficacy and safety meta-analysis of transcranial magnetic stimulation for post-stroke cognitive impairment. Chin J Rehabil Med. (2021) 36:1555–60. doi: 10.3969/j.issn.1001-1242.2021.12.013

[35] Wang RL Han ZC. Efficacy and safety of repetitive transcranial magnetic stimulation in the treatment of post-stroke cognitive impairment: a systematic review. Chin J Inter Med Cardio/Cerebrovasc Dis. (2022) 20:1379–86. doi: 10.12102/j.issn.1672-1349.2022.08.007

[36] Han K Liu J Tang Z Su W Liu Y Lu H . Effects of excitatory transcranial magnetic stimulation over the different cerebral hemispheres dorsolateral prefrontal cortex for post-stroke cognitive impairment: a systematic review and meta-analysis. Front Neurosci. (2023) 17:1102311. doi: 10.3389/fnins.2023.1102311, PMID: 37260845 PMC10228699

[37] Li K-P Sun J Wu C-Q An X Wu J-J Zheng M-X . Effects of repetitive transcranial magnetic stimulation on post-stroke patients with cognitive impairment: a systematic review and meta-analysis. Behav Brain Res. (2023) 439:114229. doi: 10.1016/j.bbr.2022.114229, PMID: 36442646

[38] Xie H Luo S Xiong D Zhu P Chen J Tang X . Efficacy and safety of repetitive transcranial magnetic stimulation for poststroke memory disorder: a meta-analysis and systematic review. J Integr Neurosci. (2023) 22:131. doi: 10.31083/j.jin2205131 37735134

[39] Chen X Liu F Lyu Z Xiu H Hou Y Tu S. High-frequency repetitive transcranial magnetic stimulation (HF-rTMS) impacts activities of daily living of patients with post-stroke cognitive impairment: a systematic review and meta-analysis. Neurol Sci. (2023) 44:2699–713. doi: 10.1007/s10072-023-06779-9, PMID: 37012519

[40] Gao Y Qiu Y Yang Q Tang S Gong J Fan H . Repetitive transcranial magnetic stimulation combined with cognitive training for cognitive function and activities of daily living in patients with post-stroke cognitive impairment: a systematic review and meta-analysis. Ageing Res Rev. (2023) 87:101919. doi: 10.1016/j.arr.2023.101919, PMID: 37004840

[41] Xu WW Liao QH Zhu DW. The effect of transcranial magnetic stimulation on the recovery of attention and memory impairment following stroke: a systematic review and meta-analysis. Expert Rev Neurother. (2022) 22:1031–41. doi: 10.1080/14737175.2022.2155515, PMID: 36469637

[42] Liu M Bao G Bai L Yu E. The role of repetitive transcranial magnetic stimulation in the treatment of cognitive impairment in stroke patients: a systematic review and meta-analysis. Sci Prog. (2021) 104:003685042110042. doi: 10.1177/00368504211004266, PMID: 33827345 PMC10455033

[43] Gong C Hu H Peng XM Li H Xiao L Liu Z . Therapeutic effects of repetitive transcranial magnetic stimulation on cognitive impairment in stroke patients: a systematic review and meta-analysis. Front Hum Neurosci. (2023) 17:1177594. doi: 10.3389/fnhum.2023.1177594, PMID: 37250691 PMC10213559

[44] Li Q Xu YL Li PT. Meta-analysis of the efficacy of high-frequency rTMS in treating post-stroke cognitive impairment in China. Yi Shou Bao Dian. (2023) 12:0047–54.

[45] Tian RH Bing YJ Li T. Clinical efficacy and safety of repetitive transcranial magnetic stimulation for cognitive impairment after stroke: a Meta-analysis. Chin Sci and Technol J Database (full text version) Med and Health Care. (2023) 12:0047–54.

[46] Hara T Shanmugalingam A McIntyre A Burhan AM. The effect of non-invasive brain stimulation (NIBS) on attention and memory function in stroke rehabilitation patients: a systematic review and Meta-analysis. Diagnostics. (2021) 11:227. doi: 10.3390/diagnostics11020227, PMID: 33546266 PMC7913379

[47] Chen X Xiu H Hou Y Chen X Liu F Tu S. High-frequency repetitive transcranial magnetic stimulation (HF-rTMS) on overall cognition in patients with post-stroke cognitive impairment: a systematic review and Meta-analysis. Am J Phys Med Rehabil. (2023). 10–1097. doi: 10.1097/PHM.0000000000002377, PMID: 38113027

[48] Guyatt GH Oxman AD Vist GE Kunz R Falck-Ytter Y Alonso-Coello P . GRADE: an emerging consensus on rating quality of evidence and strength of recommendations. Chin J Evid Based Med. (2009) 9:8–11. doi: 10.3969/j.issn.1672-2531.2009.01.005

[49] Kalaria RN Akinyemi R Ihara M. Stroke injury, cognitive impairment and vascular dementia. Biochim Biophys Acta. (2016) 1862:915–25. doi: 10.1016/j.bbadis.2016.01.015 26806700 PMC4827373

[50] Tomlinson BE Blessed G Roth M. Observations on the brains of demented old people. J Neurol Sci. (1970) 11:205–42. doi: 10.1016/0022-510x(70)90063-8 5505685

[51] Zekry D Duyckaerts C Belmin J Geoffre C Herrmann F Moulias R . The vascular lesions in vascular and mixed dementia: the weight of functional neuroanatomy. Neurobiol Aging. (2003) 24:213–9. doi: 10.1016/S0197-4580(02)00066-0, PMID: 12498955

[52] Szabo K Förster A Jäger T Kern R Griebe M Hennerici MG . Hippocampal lesion patterns in acute posterior cerebral artery stroke: clinical and MRI findings. Stroke. (2009) 40:2042–5. doi: 10.1161/STROKEAHA.108.536144, PMID: 19359650

[53] Thiel A Cechetto DF Heiss W-D Hachinski V Whitehead SN. Amyloid burden, Neuroinflammation, and links to cognitive decline after ischemic stroke. Stroke. (2014) 45:2825–9. doi: 10.1161/STROKEAHA.114.004285 25005439

[54] Cao W Cao X Hou C Li T Cheng Y Jiang L . Effects of cognitive training on resting-state functional connectivity of default mode, salience, and central executive networks. Front Aging Neurosci. (2016) 8:70. doi: 10.3389/fnagi.2016.00070, PMID: 27148042 PMC4828428

[55] Song J Kim E Kim C-H Song H-T Lee JE. The role of orexin in post-stroke inflammation, cognitive decline, and depression. Mol Brain. (2015) 8:16. doi: 10.1186/s13041-015-0106-1, PMID: 25884812 PMC4357085

[56] Narasimhalu K Lee J Leong YL Ma L De Silva DA Wong MC . Inflammatory markers and their association with post stroke cognitive decline. Int J Stroke. (2015) 10:513–8. doi: 10.1111/ijs.12001, PMID: 23489773

[57] Mok V Liu W Wong A. Detection of amyloid plaques in patients with post-stroke dementia. Hong Kong Med J. (2016) 22:S40–2. PMID: 26908343

[58] Su H Zhong N Gan H Wang J Han H Chen T . High frequency repetitive transcranial magnetic stimulation of the left dorsolateral prefrontal cortex for methamphetamine use disorders: a randomised clinical trial. Drug Alcohol Depend. (2017) 175:84–91. doi: 10.1016/j.drugalcdep.2017.01.037, PMID: 28410525

[59] Zhang XQ Li L Huo JT Cheng M Li LH. Effects of repetitive transcranial magnetic stimulation on cognitive function and cholinergic activity in the rat hippocampus after vascular dementia. Neural Regen Res. (2018) 13:1384–9. doi: 10.4103/1673-5374.235251, PMID: 30106050 PMC6108210

[60] Zhang F Qin Y Xie L Zheng C Huang X Zhang M. High-frequency repetitive transcranial magnetic stimulation combined with cognitive training improves cognitive function and cortical metabolic ratios in Alzheimer’s disease. J Neural Transm. (2019) 126:1081–94. doi: 10.1007/s00702-019-02022-y, PMID: 31292734

[61] Yuan LQ Zeng Q Wang D Wen XY Shi Y Zhu F . Neuroimaging mechanisms of high-frequency repetitive transcranial magnetic stimulation for treatment of amnestic mild cognitive impairment: a double-blind randomized sham-controlled trial. Neural Regen Res. (2021) 16:707–13. doi: 10.4103/1673-5374.295345, PMID: 33063732 PMC8067941

[62] Bashir S Al-Hussain F Hamza A Shareefi GF Abualait T Yoo WK. Role of single low pulse intensity of transcranial magnetic stimulation over the frontal cortex for cognitive function. Front Hum Neurosci. (2020) 14:205. doi: 10.3389/fnhum.2020.00205, PMID: 32719592 PMC7350777

[63] Park IS Yoon JG. The effect of computer-assisted cognitive rehabilitation and repetitive transcranial magnetic stimulation on cognitive function for stroke patients. J Phys Ther Sci. (2015) 27:773–6. doi: 10.1589/jpts.27.773, PMID: 25931728 PMC4395712

[64] Zhu H Xu G Fu L Li Y Fu R Zhao D . The effects of repetitive transcranial magnetic stimulation on the cognition and neuronal excitability of mice. Electromagn Biol Med. (2020) 39:9–19. doi: 10.1080/15368378.2019.1696358, PMID: 31762316

[65] Xu Y Qiu Z Zhu J Liu J Wu J Tao J . The modulation effect of non-invasive brain stimulation on cognitive function in patients with mild cognitive impairment: a systematic review and meta-analysis of randomized controlled trials. BMC Neurosci. (2019) 20:2. doi: 10.1186/s12868-018-0484-2 30602377 PMC6317253

[66] Ma J Wang J Lv C Pang J Han B Wang M . The role of hippocampal structural synaptic plasticity in repetitive transcranial magnetic stimulation to improve cognitive function in male SAMP8 mice. Cell Physiol Biochem. (2017) 41:137–44. doi: 10.1159/000455982, PMID: 28214838

[67] Lin Y Jiang WJ Shan PY Lu M Wang T Li RH . The role of repetitive transcranial magnetic stimulation (rTMS) in the treatment of cognitive impairment in patients with Alzheimer’s disease: a systematic review and meta-analysis. J Neurol Sci. (2019) 398:184–91. doi: 10.1016/j.jns.2019.01.038, PMID: 30735817

[68] Farokhi-Sisakht F Farhoudi M Sadigh-Eteghad S Mahmoudi J Mohaddes G. Cognitive rehabilitation improves ischemic stroke-induced cognitive impairment: role of growth factors. J Stroke Cerebrovasc Dis. (2019) 28:104299. doi: 10.1016/j.jstrokecerebrovasdis.2019.07.015, PMID: 31371141

[69] Medina-Fernández FJ Escribano BM Padilla-del-Campo C Drucker-Colín R Pascual-Leone Á Túnez I. Transcranial magnetic stimulation as an antioxidant. Free Radic Res. (2018) 52:381–9. doi: 10.1080/10715762.2018.1434313 29385851

