# Supplementary material for: Efficacy of repetitive transcranial magnetic stimulation in post-stroke cognitive impairment: an overview of systematic reviews
Source: Front Neurol. 2024 Apr 22;15:1378731. doi: 10.3389/fneur.2024.1378731 (PMC11075487; doi:10.3389/fneur.2024.1378731)
Supplement: Supplementary file 1 [file Table_1.DOCX]

Supplementary Material

**Efficacy of repetitive transcranial magnetic stimulation in Post-stroke cognitive impairment: an Overview of Systematic Reviews**

**Linli Zhang^1, 2†^, Shan Gao^2†^, Chengshuo Wang^1, 3^, Yuanyuan Li^1^, Huateng Yuan^2^, Longjun Cao^1*^, Chong Gao^2*^**

***Correspondence:**

**Longjun Cao**

longjuncao@tjus.edu.cn

**Chong Gao**

[Gaochong0005@163.com](mailto:Gaochong0005@163.com)

**^†^**These authors contributed equally to this work

# **1　　Supplementary Tables**

**Supplementary Table S1. Search strategy (****Pubmed database retrieval strategies).**

| **Database** | **Database** | **Retrieval date** |
| --- | --- | --- |
| PubMed | #1 "Stroke"[Mesh]  #2 Strokes[Title/Abstract] OR Cerebrovascular Accident[Title/Abstract] OR Cerebrovascular Accidents[Title/Abstract] OR CVA (Cerebrovascular Accident[Title/Abstract]) OR CVAs (Cerebrovascular Accident[Title/Abstract]) OR Cerebrovascular Apoplexy[Title/Abstract] OR Apoplexy, Cerebrovascular[Title/Abstract] OR Vascular Accident, Brain[Title/Abstract] OR Brain Vascular Accident[Title/Abstract] OR Brain Vascular Accidents[Title/Abstract] OR Vascular Accidents, Brain[Title/Abstract] OR Cerebrovascular Stroke[Title/Abstract] OR Cerebrovascular Strokes[Title/Abstract] OR Stroke, Cerebrovascular[Title/Abstract] OR Strokes, Cerebrovascular[Title/Abstract] OR Apoplexy[Title/Abstract] OR Cerebral Stroke[Title/Abstract] OR Cerebral Strokes[Title/Abstract] OR Stroke, Cerebral[Title/Abstract] OR Strokes, Cerebral[Title/Abstract] OR Stroke, Acute[Title/Abstract] OR Acute Stroke[Title/Abstract] OR Acute Strokes[Title/Abstract] OR Strokes, Acute[Title/Abstract] OR Cerebrovascular Accident, Acute[Title/Abstract] OR Acute Cerebrovascular Accident[Title/Abstract] OR Acute Cerebrovascular Accidents[Title/Abstract] OR Cerebrovascular Accidents, Acute[Title/Abstract]  #3 #1 OR #2  #4 "Transcranial Magnetic Stimulation"[Mesh]  #5 Magnetic Stimulation, Transcranial[Title/Abstract] OR Magnetic Stimulations, Transcranial[Title/Abstract] OR Stimulation, Transcranial Magnetic[Title/Abstract] OR Stimulations, Transcranial Magnetic[Title/Abstract] OR Transcranial Magnetic Stimulations[Title/Abstract] OR Transcranial Magnetic Stimulation, Single Pulse[Title/Abstract] OR Transcranial Magnetic Stimulation, Paired Pulse[Title/Abstract] OR Transcranial Magnetic Stimulation, Repetitive[Title/Abstract] OR repetitive transcranial magnetic stimulation[Title/Abstract] OR TMS[Title/Abstract] OR rTMS[Title/Abstract] OR magnetic stimulation[Title/Abstract] OR TBS[Title/Abstract] OR iTBS[Title/Abstract] OR cTBS[Title/Abstract] OR Theta burst stimulation[Title/Abstract] OR intermittent theta burst stimulation[Title/Abstract] OR continuous theta burst stimulation[Title/Abstract] OR non-invasive brain stimulation[Title/Abstract]  #6 #4 OR #5  #7 "Cognitive Dysfunction"[Mesh]  #8 Cognitive Dysfunctions[Title/Abstract] OR Dysfunction, Cognitive[Title/Abstract] OR Dysfunctions, Cognitive[Title/Abstract] OR Cognitive Impairments[Title/Abstract] OR Cognitive Impairment[Title/Abstract] OR Impairment, Cognitive[Title/Abstract] OR Impairments, Cognitive Cognitive Disorder[Title/Abstract] OR Cognitive Disorders[Title/Abstract] OR Disorder, Cognitive[Title/Abstract] OR Disorders, Cognitive[Title/Abstract] OR Mild Cognitive Impairment[Title/Abstract] OR Cognitive Impairment, Mild[Title/Abstract] OR Cognitive Impairments, Mild[Title/Abstract] OR Impairment, Mild Cognitive[Title/Abstract] OR Impairments, Mild Cognitive[Title/Abstract] OR Mild Cognitive Impairments[Title/Abstract] OR Cognitive Decline[Title/Abstract] OR Cognitive Declines[Title/Abstract] OR Decline, Cognitive[Title/Abstract] OR Declines, Cognitive[Title/Abstract] OR Mental Deterioration[Title/Abstract] OR Deterioration, Mental[Title/Abstract] OR Deteriorations, Mental[Title/Abstract] OR Mental Deteriorations[Title/Abstract] OR Post-stroke cognitive dysfunction[Title/Abstract]  #9 #7 or #8  #10 "Systematic Review"[Publication Type]  #11 "Meta-Analysis"[Publication Type]  #12 meta-analysis[Title/Abstract] OR meta analysis[Title/Abstract] OR systematic review[Title/Abstract] OR review Overview, Clinical Trial[Title/Abstract] OR Clinical Trial Overview[Title/Abstract]  #13 #10 OR #11 OR #12  #14 #3 and #6 and #9 and #13 | March 26, 2024 |

**Supplementary Table S2. Search strategy (Common database retrieval strategies).**

| **Database** | **Database** | **Retrieval date** |
| --- | --- | --- |
| Embase | #1 'cerebrovascular accident'/exp  #2 'strokes':ab,ti OR 'cerebrovascular accident':ab,ti OR 'cerebrovascular accidents':ab,ti OR'cva (cerebrovascular accident)':ab,ti OR 'cvas (cerebrovascular accident)':ab,ti OR 'cerebrovascular apoplexy':ab,ti OR 'apoplexy, cerebrovascular':ab,ti OR 'vascular accident, brain':ab,ti OR 'brain vascular accident':ab,ti OR 'brain vascular accidents':ab,ti OR 'vascular accidents, brain':ab,ti OR 'cerebrovascular stroke':ab,ti OR 'cerebrovascular strokes':ab,ti OR 'stroke,cerebrovascular':ab,ti OR 'strokes, cerebrovascular':ab,ti OR 'apoplexy':ab,ti OR 'cerebral stroke':ab,ti OR 'cerebral strokes':ab,ti OR 'stroke, cerebral':ab,ti OR 'strokes, cerebral':ab,ti OR 'stroke, acute':ab,ti OR 'acute stroke':ab,ti OR 'acute strokes':ab,ti OR 'strokes, acute':ab,ti OR 'cerebrovascular accident, acute':ab,ti OR 'acute cerebrovascular accident':ab,ti OR 'acute cerebrovascular accidents':ab,ti OR 'cerebrovascular':ab,ti  #3 'repetitive transcranial magnetic stimulation'/exp  #4 'transcranial magnetic stimulation':ab,ti OR 'magnetic stimulation, transcranial':ab,ti OR 'magnetic stimulations, transcranial':ab,ti OR 'stimulation, transcranial magnetic':ab,ti OR 'stimulations, transcranial magnetic':ab,ti OR 'transcranial magnetic stimulations':ab,ti OR 'transcranial magnetic stimulation, single pulse':ab,ti OR 'transcranial magnetic stimulation, paired pulse':ab,ti OR 'transcranial magnetic stimulation, repetitive':ab,ti OR 'tms':ab,ti OR 'rtms':ab,ti OR 'magnetic stimulation':ab,ti OR 'tbs':ab,ti OR 'itbs':ab,ti OR 'ctbs':ab,ti OR 'theta burst stimulation':ab,ti OR 'intermittent theta burst stimulation':ab,ti OR 'continuous theta burst stimulation':ab,ti OR 'non-invasive brain stimulation':ab,ti  #5 'cognitive defect'/exp  #6 'cognitive dysfunction':ab,ti OR 'cognitive dysfunctions':ab,ti OR 'dysfunction, cognitive':ab,ti OR 'dysfunctions, cognitive':ab,ti OR 'cognitive impairments':ab,ti OR 'cognitive impairment':ab,ti OR 'impairment, cognitive':ab,ti OR 'impairments, cognitive cognitive disorder':ab,ti OR cognitive disorders':ab,ti OR 'disorder, cognitive':ab,ti OR'disorders, cognitive':ab,ti OR 'mild cognitive impairment':ab,ti OR 'cognitive impairment, mild':ab,ti OR 'cognitive impairments, mild':ab,ti OR 'impairment, mild cognitive':ab,ti OR 'impairments, mild cognitive':ab,ti OR 'mild cognitive impairments':ab,ti OR ab,ti OR 'cognitive decline':ab,ti OR 'cognitive declines':ab,ti OR 'decline, cognitive':ab,ti OR 'declines, cognitive':ab,ti OR 'mental deterioration':ab,ti OR 'deterioration, menta':ab,ti OR 'deteriorations, mental':ab,ti OR 'mental deteriorations':ab,ti OR 'post-stroke cognitive dysfunction':ab,ti  #7 'systematic review'/exp  #8 'meta analysis'/exp  #9 'meta-analysis':ab,ti OR 'meta analysis':ab,ti OR 'systematic review':ab,ti OR 'review overview, clinical trial':ab,ti OR 'clinical trial overview':ab,ti  #10 #1 or #2  #11 #3 or #4  #12 #5 or #6  #13 #7 or #8 or #9  #14 #10 and #11 and #12 and #13 | March 26, 2024 |
| Cochrane Library | #1 MeSH descriptor: [Stroke] explode all trees  #2 (Strokes):ab,ti,kw or (Cerebrovascular Accident):ab,ti,kw or (Cerebrovascular Accidents):ab,ti,kw or (CVA (Cerebrovascular Accident)):ab,ti,kw or (CVAs (Cerebrovascular Accident)):ab,ti,kw or (Cerebrovascular Apoplexy):ab,ti,kw or (Apoplexy, Cerebrovascular):ab,ti,kw or (Vascular Accident, Brain):ab,ti,kw or (Brain Vascular Accident):ab,ti,kw or (Brain Vascular Accidents):ab,ti,kw or (Vascular Accidents, Brain):ab,ti,kw or ( Cerebrovascular Stroke):ab,ti,kw or (Cerebrovascular Strokes):ab,ti,kw or (Stroke, Cerebrovascular):ab,ti,kw or (Strokes, Cerebrovascular):ab,ti,kw or (Apoplexy):ab,ti,kw or (Cerebral Stroke):ab,ti,kw or (Cerebral Strokes):ab,ti,kw or (Stroke, Cerebral):ab,ti,kw or (Strokes, Cerebral):ab,ti,kw or (Stroke, Acute):ab,ti,kw or ( Acute Stroke):ab,ti,kw or (Acute Strokes):ab,ti,kw or (Strokes, Acute):ab,ti,kw or (Cerebrovascular Accident, Acute):ab,ti,kw or (Acute Cerebrovascular Accident):ab,ti,kw or (Acute Cerebrovascular Accidents):ab,ti,kw or ( Cerebrovascular Accidents, Acute):ab,ti,kw  #3 MeSH descriptor: [Transcranial Magnetic Stimulation] explode all trees  #4 (Magnetic Stimulation, Transcranial):ti,ab,kw or (Magnetic Stimulations, Transcranial):ti,ab,kw or (Stimulation, Transcranial Magnetic):ti,ab,kw or (Stimulations, Transcranial Magnetic):ti,ab,kw or (Transcranial Magnetic Stimulations):ti,ab,kw or (Transcranial Magnetic Stimulation, Single Pulse):ti,ab,kw or (Transcranial Magnetic Stimulation, Paired Pulse):ti,ab,kw or (Transcranial Magnetic Stimulation, Repetitive):ti,ab,kw or (repetitive transcranial magnetic stimulation):ti,ab,kw or (TMS):ti,ab,kw or (rTMS):ti,ab,kw or (magnetic stimulation):ti,ab,kw or (TBS):ti,ab,kw or (iTBS):ti,ab,kw or (cTBS):ti,ab,kw or (Theta burst stimulation):ti,ab,kw or (intermittent theta burst stimulation):ti,ab,kw or (continuous theta burst stimulation):ti,ab,kw or (non-invasive brain stimulation): ti,ab,kw  #5 MeSH descriptor: [Meta-Analysis as Topic] explode all trees  #6 MeSH descriptor: [Systematic Reviews as Topic] explode all trees  #7 (meta-analysis):ti,ab,kw or (meta analysis):ti,ab,kw or (systematic review):ti,ab,kw or (review overview, clinical trial):ti,ab,kw or (clinical trial overview):ti,ab,kw  #8 MeSH descriptor: [Cognitive Dysfunction] explode all trees  #9 (Cognitive Dysfunctions):ti,ab,kw or (Dysfunction, Cognitive):ti,ab,kw or (Dysfunctions, Cognitive):ti,ab,kw or (Cognitive Impairments):ti,ab,kw or (Cognitive Impairment):ti,ab,kw or (Impairment, Cognitive):ti,ab,kw or (Impairments, Cognitive Cognitive Disorder):ti,ab,kw or (Cognitive Disorders):ti,ab,kw or (Disorder, Cognitive):ti,ab,kw or (Disorders, Cognitive):ti,ab,kw or (Mild Cognitive Impairment):ti,ab,kw or (Cognitive Impairment, Mild):ti,ab,kw or (Cognitive Impairments, Mild):ti,ab,kw or (Impairment, Mild Cognitive):ti,ab,kw or (Impairments, Mild Cognitive):ti,ab,kw or (Mild Cognitive Impairments):ti,ab,kw or (Cognitive Decline):ti,ab,kw or (Cognitive Declines):ti,ab,kw or (Decline, Cognitive):ti,ab,kw or (Declines, Cognitive):ti,ab,kw or (Mental Deterioration):ti,ab,kw or (Deterioration, Mental):ti,ab,kw or (Deteriorations, Mental):ti,ab,kw or (Mental Deteriorations):ti,ab,kw or (Post-stroke cognitive dysfunction):ti,ab,kw  #10 #1 or #2  #11 #3 or #4  #12 #5 or #6 or #7  #13 #8 or #9  #14 #10 and #11 and #12 and #13 | March 26, 2024 |
| Web of Science | #1 TS=(Strokes or Cerebrovascular Accident or Cerebrovascular Accidents or CVA (Cerebrovascular Accident) or CVAs (Cerebrovascular Accident) or Cerebrovascular Apoplexy or Apoplexy, Cerebrovascular or Vascular Accident, Brain or Brain Vascular Accident or Brain Vascular Accidents or Vascular Accidents, Brain or Cerebrovascular Stroke or Cerebrovascular Strokes or Stroke, Cerebrovascular or Strokes, Cerebrovascular or Apoplexy or Cerebral Stroke or Cerebral Strokes or Stroke, Cerebral or Strokes, Cerebral or Stroke, Acute or Acute Stroke or Acute Strokes or Strokes, Acute or Cerebrovascular Accident, Acute or Acute Cerebrovascular Accident or Acute Cerebrovascular Accidents or Cerebrovascular Accidents, Acute)  #2 TS=(transcranial magnetic stimulation or Magnetic Stimulation, Transcranial or Magnetic Stimulations, Transcranial or Stimulation, Transcranial Magnetic or Stimulations, Transcranial Magnetic or Transcranial Magnetic Stimulations or Transcranial Magnetic Stimulation, Single Pulse or Transcranial Magnetic Stimulation, Paired Pulse or Transcranial Magnetic Stimulation, Repetitive or TMS or rTMS or magnetic stimulation or TBS or iTBS or cTBS or theta burst stimulation or intermittent theta burst stimulation or continuous theta burst stimulation or non-invasive brain stimulation)  #3 TS=(meta-analysis or meta analysis or systematic review or review Overview, Clinical Trial or Clinical Trial Overview)  #4 TS=(Cognitive Dysfunction or Cognitive Dysfunctions or Dysfunction, Cognitive or Dysfunctions, Cognitive or Cognitive Impairments or Cognitive Impairment or Impairment, Cognitive or Impairments, Cognitive Cognitive Disorder or Cognitive Disorders or Disorder, Cognitive or Disorders, Cognitive or Mild Cognitive Impairment or Cognitive Impairment, Mild or Cognitive Impairments, Mild or Impairment, Mild Cognitive or Impairments, Mild Cognitive or Mild Cognitive Impairments or Cognitive Decline or Cognitive Declines or Decline, Cognitive or Declines, Cognitive or Mental Deterioration or Deterioration, Mental or Deteriorations, Mental or Mental Deteriorations OR Post-stroke cognitive dysfunction)  #5 #1 and #2 and #3 and #4 | March 26, 2024 |
| 中国知网(CNKI) | #1 主题：脑卒中 + 脑出血 + 脑血管意外 + 卒中 + 中风 + 脑梗塞  #2 经颅磁刺激 + rTMS + TMS + TBS + iTBS + cTBS + 重复经颅磁刺激 + 磁刺激 + 连续性θ节律性磁刺激 + 间歇性θ节律刺激 + θ刺激 + theta节律刺激 + 非入侵性脑刺激  #3 认知障碍 + 认知功能 + 记忆 + 执行功能 + 工作记忆 + 注意力  #4 系统评价 + meta分析 + 荟萃分析 + 元分析  #5 #1 and #2 and #3 and #4 | March 26, 2024 |
| 万方数据 | #1 主题或关键词：脑卒中 OR 脑出血 OR 脑血管意外 OR 卒中 OR 中风  #2 主题或关键词：经颅磁刺激 OR rTMS OR TMS OR TBS OR iTBS OR cTBS OR 重复经颅磁刺激 OR 磁刺激 OR 连续性θ节律性磁刺激 OR 间歇性θ节律刺激 OR θ刺激 OR theta节律刺激 OR 非入侵性脑刺激  #3 主题或关键词：认知障碍 OR 认知功能 OR 记忆 OR 执行功能 OR 工作记忆 OR 注意力  #4 主题或关键词：系统评价 OR meta分析 OR 荟萃分析 OR 元分析  #5 #1 与 #2 与 #3 与 #4 | March 26, 2024 |
| 维普 | #1 主题或关键词：脑卒中 OR 脑出血 OR 脑血管意外 OR 卒中 OR 中风  #2 主题或关键词：经颅磁刺激 OR 重复经颅磁刺激 OR 磁刺激 OR 连续性θ节律性磁刺激 OR 间歇性θ节律刺激 OR θ刺激 OR theta节律刺激 OR 非入侵性脑刺激 OR rTMS OR TMS OR TBS OR iTBS  #3 主题或关键词：认知障碍 OR 认知功能 OR 记忆 OR 执行功能 OR 工作记忆 OR 注意力  #4 主题或关键词：系统评价 OR meta分析 OR 荟萃分析 OR 元分析  #5 #1 与 #2 与 #3 与 #4 | March 26, 2024 |
| 中国生物医学文献数据库(CBM) | 1) "卒中"[不加权:扩展]  2) "脑出血"[常用字段:智能] OR "脑血管意外"[常用字段:智能] OR "脑梗塞"[常用字段:智能] OR "卒中"[常用字段:智能] OR "中风"[常用字段:智能]  3) "经颅磁刺激"[不加权:扩展]  4) "重复经颅磁刺激"[常用字段:智能] OR "rTMS"[常用字段:智能] OR "磁刺激"[常用字段:智能] OR "连续性θ节律性磁刺激"[常用字段:智能] OR "间歇性θ节律刺激"[常用字段:智能] OR "θ刺激"[常用字段:智能] OR "theta节律刺激"[常用字段:智能] OR "非入侵性脑刺激"[常用字段:智能] OR "rTMS"[常用字段:智能] OR "TMS"[常用字段:智能] OR "TBS"[常用字段:智能] OR "iTBS"[常用字段:智能]  5) "认知障碍"[不加权:扩展]  6) "认知功能"[常用字段:智能] OR "记忆"[常用字段:智能] OR "执行功能"[常用字段:智能] OR "工作记忆"[常用字段:智能] OR "注意力"[常用字段:智能]  7) "系统评价(主题)"[不加权:扩展]  8) "Meta分析"[不加权:扩展]  9) "系统评价"[常用字段:智能] OR "meta分析"[常用字段:智能] OR "荟萃分析"[常用字段:智能] OR "元分析"[常用字段:智能]  10) (#2) OR (#1)  11) (#4) OR (#3)  12) (#6) OR (#5)  13) (#9) OR (#8) OR (#7)  14) (#13) AND (#12) AND (#11) AND (#10) | March 26, 2024 |

**Supplementary Table S3. AMSTAR-2 scale included MAs/SRs.**

| Study ID | 1 | 2* | 3 | 4* | 5 | 6 | 7* | 8 | 9* | 10 | 11* | 12 | 13* | 14 | 15* | 16 | Quality grade |
| --- | --- | --- | --- | --- | --- | --- | --- | --- | --- | --- | --- | --- | --- | --- | --- | --- | --- |
| Sun et al. (31) | Y | N | N | PY | Y | N | N | PY | Y | N | Y | N | N | Y | N | N | Very low |
| Yin et al. (32) | Y | N | N | PY | Y | Y | N | PY | Y | N | Y | N | N | Y | Y | Y | Very low |
| Liu et al. (33) | Y | N | N | PY | Y | Y | N | PY | Y | N | N | N | N | N | N | N | Very low |
| Zhu et all. (34) | Y | N | N | PY | Y | Y | N | PY | Y | N | Y | N | N | Y | Y | N | Very low |
| Wang and Han. (35) | Y | N | N | PY | Y | Y | N | PY | Y | N | N | N | N | N | Y | N | Very low |
| Han et al. (36) | Y | Y | N | PY | Y | Y | N | PY | Y | N | Y | Y | N | Y | N | Y | Very low |
| Li et al. (37) | Y | Y | N | PY | N | Y | N | PY | Y | N | Y | Y | N | N | Y | Y | Very low |
| Xie et al. (38) | Y | Y | N | PY | Y | Y | N | PY | Y | N | Y | Y | N | Y | Y | Y | Very low |
| Chen et al. (39) | Y | N | N | PY | Y | N | N | PY | Y | N | Y | N | N | Y | N | Y | Very low |
| Gao et al. (40) | Y | Y | N | PY | Y | Y | N | PY | Y | N | Y | N | Y | Y | N | Y | Very low |
| Xu et al. (41) | Y | N | N | PY | N | Y | N | PY | PY | N | Y | Y | N | N | N | Y | Very low |
| Liu et al. (42) | Y | N | N | PY | N | Y | N | PY | Y | N | N | N | N | N | N | Y | Very low |
| Gong et al. (43) | Y | Y | N | PY | Y | Y | N | PY | Y | N | N | N | N | N | N | Y | Very low |
| Li et al. (44) | Y | N | N | PY | N | N | N | PY | Y | N | Y | N | N | N | N | N | Very low |
| Tian et al. (45) | Y | N | N | PY | Y | Y | N | PY | Y | N | N | N | N | N | Y | N | Very low |
| Hara et al. (46) | Y | Y | N | PY | Y | Y | N | PY | Y | N | Y | N | Y | N | Y | Y | Very low |
| Chen et al. (47) | Y | Y | N | PY | Y | Y | N | PY | Y | N | Y | Y | N | Y | N | Y | Very low |
| Reporting rate (%) | 100 | 41 | 0 | 100 | 76 | 82 | 0 | 100 | 100 | 0 | 71 | 29 | 12 | 47 | 41 | 65 | — |

item 1 Do the study questions and inclusion criteria include PICO? item 2: Is there a pre-published protocol? Is there significant bias between the study and the protocol? item 3: Did the authors explain the type of study design included? item 4: Was a comprehensive literature search strategy used? item 5: Was duplicate study screening performed? item 6: Were duplicate data extractions performed? item 7: Was a list of excluded literature provided, with reasons for the exclusion? item 8: Was a detailed description of the included studies provided? item 9: Was the risk of bias for each included study assessed using a reasonable tool? item 10: Is the source of funds for the included studies reported? item 11: If Meta-analyses were performed, were the results statistically combined using appropriate methods? item 12: If Meta-analyses were performed, is the effect of risk of bias described in the results? item 13: If a Meta-analysis was performed, is the effect of risk of bias described in the discussion? item 14: Is heterogeneity justified in the discussion? item 15: If a quantitative analysis was performed, was publication bias adequately investigated and its possible impact discussed? item 16: Are any potential sources of conflict of interest reported?

* represents the key items in the AMSTAR 2 scale;Y = Yes; PY = Partial Yes; N = No;

Item reporting rate = [(number of studies with full reporting of entries + number of studies with partial reporting)/total number of included studies] × 100%.

**Supplementary Table S4　PRISMA declaration entries report conditions.**

| Stud ID | 1 | 2 | 3 | 4 | 5 | 6 | 7 | 8 | 9 | 10 | 11 | 12 | 13 | 14 | 15 | 16 |
| --- | --- | --- | --- | --- | --- | --- | --- | --- | --- | --- | --- | --- | --- | --- | --- | --- |
| Sun et al. (31) | 1 | 0.5 | 1 | 1 | 0.5 | 0.5 | 0.5 | 1 | 0.5 | 0.5 | 1 | 1 | 0.5 | 1 | 0 | 1 |
| Yin et al. (32) | 1 | 0.5 | 1 | 1 | 0.5 | 0.5 | 0.5 | 1 | 1 | 0.5 | 1 | 1 | 1 | 1 | 1 | 1 |
| Liu et al. (33) | 1 | 0.5 | 1 | 1 | 0.5 | 0.5 | 0 | 1 | 0.5 | 0.5 | 0.5 | 1 | 0.5 | 0 | 0 | 0.5 |
| Zhu et all. (34) | 1 | 0 | 1 | 1 | 1 | 0.5 | 0 | 1 | 1 | 0.5 | 0.5 | 1 | 0.5 | 1 | 0 | 1 |
| Wang and Han. (35) | 1 | 0.5 | 1 | 1 | 0.5 | 0.5 | 0 | 1 | 0.5 | 0.5 | 0.5 | 1 | 0.5 | 1 | 0 | 1 |
| Han et al. (36) | 1 | 1 | 1 | 1 | 0.5 | 0.5 | 0.5 | 1 | 1 | 0.5 | 1 | 1 | 0.5 | 0 | 1 | 1 |
| Li et al. (37) | 1 | 0.5 | 1 | 1 | 0.5 | 0.5 | 0 | 0.5 | 1 | 0.5 | 1 | 1 | 0.5 | 1 | 0 | 1 |
| Xie et al. (38) | 1 | 0.5 | 1 | 1 | 1 | 1 | 1 | 1 | 1 | 0.5 | 1 | 1 | 1 | 1 | 1 | 1 |
| Chen et al. (39) | 1 | 0.5 | 1 | 1 | 0.5 | 0.5 | 0 | 1 | 0 | 0.5 | 1 | 1 | 1 | 0 | 1 | 1 |
| Gao et al. (40) | 1 | 0.5 | 1 | 1 | 0.5 | 0.5 | 1 | 1 | 1 | 0.5 | 1 | 1 | 0.5 | 0 | 1 | 1 |
| Xu et al. (41) | 1 | 0.5 | 1 | 1 | 1 | 0.5 | 0 | 0 | 1 | 0.5 | 1 | 1 | 0.5 | 0 | 0 | 1 |
| Liu et al. (42) | 1 | 0.5 | 1 | 1 | 0.5 | 0.5 | 0 | 0 | 1 | 0.5 | 0.5 | 1 | 0.5 | 0 | 1 | 1 |
| Gong et al. (43) | 1 | 0.5 | 1 | 1 | 1 | 0.5 | 0.5 | 1 | 1 | 0.5 | 1 | 1 | 0.5 | 0 | 1 | 1 |
| Li et al. (44) | 1 | 0.5 | 1 | 1 | 0.5 | 0.5 | 0 | 0 | 0 | 0.5 | 0.5 | 1 | 0.5 | 0 | 0 | 0.5 |
| Tian et al. (45) | 1 | 0.5 | 1 | 1 | 0.5 | 0.5 | 0.5 | 1 | 0.5 | 0.5 | 0.5 | 1 | 0.5 | 1 | 0 | 1 |
| Hara et al. (46) | 1 | 1 | 1 | 1 | 0.5 | 0.5 | 0.5 | 1 | 0.5 | 0.5 | 1 | 1 | 0.5 | 1 | 0 | 0.5 |
| Chen et al. (47) | 1 | 1 | 1 | 1 | 0.5 | 1 | 1 | 1 | 1 | 0.5 | 1 | 1 | 0.5 | 0 | 0 | 1 |
| Reporting rate (%) | 100 | 56 | 100 | 100 | 62 | 56 | 35 | 79 | 74 | 50 | 82 | 100 | 59 | 47 | 41 | 91 |

**SupplementaryTable S4 (Continued).**

| Study ID | 17 | 18 | 19 | 20 | 21 | 22 | 23 | 24 | 25 | 26 | 27 | Total Score |
| --- | --- | --- | --- | --- | --- | --- | --- | --- | --- | --- | --- | --- |
| Sun et al. (31) | 1 | 1 | 1 | 1 | 1 | 0 | 0.5 | 0 | 1 | 0 | 0 | 18 |
| Yin et al. (32) | 1 | 1 | 1 | 1 | 1 | 1 | 0.5 | 0 | 1 | 1 | 0 | 22 |
| Liu et al. (33) | 1 | 0 | 0 | 0.5 | 0 | 0 | 0.5 | 0 | 1 | 0 | 0 | 12 |
| Zhu et all. (34) | 1 | 1 | 1 | 0.5 | 1 | 0 | 0.5 | 0 | 1 | 0 | 0 | 17 |
| Wang and Han. (35) | 1 | 1 | 1 | 0.5 | 1 | 0 | 0.5 | 0 | 1 | 0 | 0 | 16.5 |
| Han et al. (36) | 1 | 1 | 1 | 1 | 0 | 1 | 0.5 | 0.5 | 1 | 1 | 0 | 20.5 |
| Li et al. (37) | 1 | 1 | 1 | 0.5 | 1 | 0 | 1 | 0.5 | 1 | 1 | 1 | 20 |
| Xie et al. (38) | 1 | 1 | 1 | 1 | 1 | 1 | 1 | 0.5 | 1 | 1 | 1 | 25.5 |
| Chen et al. (39) | 1 | 1 | 1 | 1 | 1 | 1 | 1 | 0 | 1 | 1 | 0 | 20 |
| Gao et al. (40) | 1 | 1 | 1 | 1 | 0 | 1 | 1 | 0.5 | 1 | 1 | 0 | 21 |
| Xu et al. (41) | 1 | 1 | 0.5 | 1 | 1 | 0 | 0.5 | 0 | 1 | 1 | 0 | 17 |
| Liu et al. (42) | 1 | 1 | 1 | 0.5 | 0 | 1 | 0.5 | 0 | 1 | 1 | 0 | 17 |
| Gong et al. (43) | 1 | 1 | 1 | 0.5 | 0 | 1 | 0.5 | 0.5 | 1 | 1 | 0 | 20 |
| Li et al. (44) | 1 | 0 | 0.5 | 0.5 | 0 | 0 | 0.5 | 0 | 0 | 0 | 0 | 10 |
| Tian et al. (45) | 1 | 1 | 1 | 0.5 | 1 | 0 | 0.5 | 0 | 0 | 0 | 0 | 16 |
| Hara et al. (46) | 1 | 1 | 1 | 0.5 | 0 | 0 | 1 | 0.5 | 1 | 1 | 1 | 19.5 |
| Chen et al. (47) | 1 | 1 | 1 | 0.5 | 0 | 0 | 0.5 | 0.5 | 1 | 1 | 0 | 19 |
| Reporting rate (%) | 100 | 88 | 88 | 71 | 53 | 41 | 65 | 21 | 88 | 65 | 18 | — |

— denotes no relevant results.

**Supplementary Table S5 GRADE classification of the included literatures for systematic review.**

| Study ID | Classification of outcomes indicators | Outcomes (number of studies) | Risk of bias | Inconsistency | Indirectness | Imprecision | Publication bias | Quality of evidence |
| --- | --- | --- | --- | --- | --- | --- | --- | --- |
| Sun et al. (31) | Global cognition | MoCA (published in 2018) (2) | -1^①^ | 0 | 0 | -1^⑤^ | 0 | Low |
|  |  | MoCA (published in 2019) (2) | -1^①^ | 0 | 0 | -1^⑤^ | 0 | Low |
|  |  | MoCA (published in 2020) (3) | -1^①^ | -2^③^ | 0 | 0 | 0 | Very low |
|  |  | MMSE (6) | -1^①^ | -2^③^ | 0 | 0 | 0 | Very low |
|  | ADL | MBI (5) | -1^①^ | 0 | 0 | -1^⑤^ | 0 | Low |
| Yin et al. (32) | Global cognition | MoCA (HF-rTMS) (9) | -1^①^ | -1^②^ | 0 | 0 | 0 | Low |
|  |  | MoCA (LF-rTMS) (4) | -1^①^ | 0 | 0 | -1^⑤^ | 0 | Low |
|  |  | MMSE (HF-rTMS) (4) | -1^①^ | -1^②^ | 0 | -1^⑤^ | 0 | Very low |
|  |  | MMSE (LF-rTMS) (2) | -1^①^ | -1^②^ | 0 | -1^⑤^ | 0 | Very low |
|  | ADL | MBI (HF-rTMS) (2) | -1^①^ | -1^②^ | 0 | -1^④⑤^ | 0 | Very low |
|  |  | MBI (LF-rTMS) (2) | -1^①^ | 0 | 0 | -1^④⑤^ | 0 | Low |
|  | Cognitive deterioration | P300 latency (HF-rTMS) (2) | -1^①^ | -1^②^ | 0 | -1^④⑤^ | 0 | Very low |
|  |  | P300 amplitude (HF-rTMS) (2) | -1^①^ | 0 | 0 | -1^⑤^ | 0 | Low |
| Liu et al. (33) | Global cognition | MoCA (11) | -1^①^ | 0 | 0 | 0 | 0 | Moderate |
|  |  | MoCA (affected DLPFC) | -1^①^ | -1^②^ | 0 | 0 | -1^⑥^ | Very low |
|  |  | MoCA (left DLPFC) | -1^①^ | 0 | 0 | 0 | -1^⑥^ | Low |
|  |  | MMSE (3) | -1^①^ | -2^③^ | 0 | 0 | -1^⑥^ | Very low |
|  | ADL | MBI (5) | -1^①^ | 0 | 0 | 0 | 0 | Moderate |
|  | Cognitive deterioration | P300 latency (4) | -1^①^ | -1^②^ | 0 | 0 | 0 | Low |
|  |  | P300 amplitude (4) | -1^①^ | 0 | 0 | 0 | 0 | Moderate |
| Zhu et al. (34) | Global cognition | MoCA (13) | -1^①^ | 0 | 0 | 0 | 0 | Moderate |
|  |  | MMSE (5) | -1^①^ | -2^③^ | 0 | -1^④^ | 0 | Very low |
|  | Memory | RBMT (5) | -1^①^ | 0 | 0 | 0 | 0 | Moderate |
|  | Executive function | LOTCA (3) | -1^①^ | -1^②^ | 0 | -1^④⑤^ | 0 | Very low |
|  | ADL | MBI (12) | -1^①^ | -2^③^ | 0 | -1^④^ | 0 | Very low |
|  | Cognitive deterioration | P300 latency (3) | -1^①^ | 0 | 0 | -1^④⑤^ | 0 | Low |
|  |  | P300 amplitude (3) | -1^①^ | -1^②^ | 0 | 0 | 0 | Low |
| Wang and Han. (35) | Global cognition | MoCA (14) | -1^①^ | 0 | 0 | 0 | -1^⑥^ | Low |
|  | ADL | MBI (5) | -1^①^ | 0 | 0 | -1^④^ | -1^⑥^ | Very low |
|  | Cognitive deterioration | P300 latency (8) | -1^①^ | 0 | 0 | -1^④^ | -1^⑥^ | Low |
|  |  | P300 amplitude (7) | -1^①^ | 0 | 0 | 0 | -1^⑥^ | Low |
| Han et al. (36) | Global cognition | MoCA (L-DLPFC) (11) | -1^①^ | -2^③^ | 0 | 0 | 0 | Very low |
|  |  | MMSE (L-DLPFC) (6) | -1^①^ | 0 | 0 | -1^⑤^ | 0 | Low |
|  | Memory | RBMT (2) | -1^①^ | 0 | 0 | -1^⑤^ | 0 | Low |
|  | Working memory | TMT-A (2) | -1^①^ | -2^③^ | 0 | -1^⑤^ | 0 | Very low |
|  |  | DS (2) | -1^①^ | 0 | 0 | -1^⑤^ | 0 | Low |
|  | Execution | SCWT-C (2) | -1^①^ | 0 | 0 | -1^⑤^ | 0 | Low |
|  |  | SCWT-Cs (2) | -1^①^ | 0 | 0 | -1^⑤^ | 0 | Low |
|  | ADL | MBI (L-DLPFC) (6) | -1^①^ | -1^②^ | 0 | -1^⑤^ | 0 | Very low |
|  |  | FIM (2) | -1^①^ | -2^③^ | 0 | -1^⑤^ | 0 | Very low |
|  | Cognitive deterioration | P300 latency (4) | -1^①^ | -1^②^ | 0 | 0 | 0 | Very low |
|  | Depression | BDI (2) | -1^①^ | 0 | 0 | -1^⑤^ | 0 | Low |
| Li et al. (37) | Global cognition | MoCA (5) | -1^①^ | 0 | 0 | -1^⑤^ | 0 | Low |
|  |  | MMSE (4) | -1^①^ | -1^②^ | 0 | -1^⑤^ | 0 | Very low |
|  |  | MoCA (HF-rTMS) (3) | -1^①^ | 0 | 0 | -1^⑤^ | 0 | Low |
|  |  | MoCA (LF-rTMS) (2) | -1^①^ | -1^②^ | 0 | -1^⑤^ | 0 | Very low |
|  |  | MMSE (HF-rTMS) (4) | -1^①^ | -2^③^ | 0 | -1^⑤^ | 0 | Very low |
|  |  | MoCA (L-DLPFC) (3) | -1^①^ | 0 | 0 | -1^⑤^ | 0 | Low |
|  |  | MMSE (L-DLPFC) (3) | -1^①^ | 0 | 0 | -1^⑤^ | 0 | Low |
|  | Memory | RBMT (3) | -1^①^ | 0 | 0 | -1^⑤^ | 0 | Low |
|  | Working memory | DST (2) | -1^①^ | 0 | 0 | -1^⑤^ | 0 | Low |
|  |  | DSF (5) | -1^①^ | -2^③^ | 0 | -1^⑤^ | 0 | Very low |
|  |  | DSB (5) | -1^①^ | -2^③^ | 0 | -1^⑤^ | 0 | Very low |
|  | ADL | MBI (5) | -1^①^ | 0 | 0 | -1^④⑤^ | 0 | Low |
|  | Depression | BDI (3) | -1^①^ | -1^②^ | 0 | -1④⑤ | 0 | Very low |
| Xie et al. (38) | Global cognition | MoCA (4) | -1^①^ | -1^②^ | 0 | -1^⑤^ | 0 | Very low |
|  | Memory | RBMT (5) | -1^①^ | 0 | 0 | -1^⑤^ | 0 | Low |
|  | ADL | MBI (3) | -1^①^ | 0 | 0 | -1^④⑤^ | 0 | Low |
| Chen et al. (39) | ADL | BI (10) | -1^①^ | -1^②^ | 0 | 0 | 0 | Low |
|  |  | BI (4 weeks) (5) | -1^①^ | 0 | 0 | 0 | 0 | Moderate |
|  |  | BI (8 weeks) (3) | -1^①^ | 0 | 0 | -1^④^ | 0 | Low |
|  |  | MBI (21) | -1^①^ | -2^③^ | 0 | 0 | 0 | Very low |
|  |  | MBI (3 weeks) (2) | -1^①^ | -2^③^ | 0 | -1^④⑤^ | 0 | Very low |
|  |  | MBI (4 weeks) (10) | -1^①^ | -1^②^ | 0 | 0 | 0 | Low |
|  |  | MBI (2 weeks) (3) | -1^①^ | 0 | 0 | -1^④⑤^ | 0 | Low |
|  |  | MBI (8 weeks) (5) | -1^①^ | 0 | 0 | 0 | 0 | Moderate |
|  |  | FIM (2) | -1^①^ | 0 | 0 | -1^⑤^ | 0 | Low |
| Gao et al. (40) | Global cognition | MoCA (4) | -1^①^ | 0 | 0 | 0 | 0 | High |
|  |  | MMSE (3) | -1^①^ | -1^②^ | 0 | 0 | 0 | Low |
|  | Attention | Auditory CPT (2) | -1^①^ | 0 | 0 | -1^⑤^ | 0 | Low |
|  |  | Visual CPT (2) | --1^①^ | 0 | 0 | -1^⑤^ | 0 | Low |
|  | Executive function | Word of color word test (2) | -1^①^ | 0 | 0 | -1^⑤^ | 0 | Low |
|  |  | Tower of London test (2) | -1^①^ | 0 | 0 | -1^⑤^ | 0 | Low |
|  |  | Color of color word test (2) | -1^①^ | 0 | 0 | -1^⑤^ | 0 | Low |
|  |  | VST-C time (2) | -1^①^ | 0 | 0 | -1^⑤^ | 0 | Low |
|  |  | VST-C error words (2) | -1^①^ | 0 | 0 | -1^⑤^ | 0 | Low |
|  |  | LOTCA (2) | -1^①^ | 0 | 0 | -1^⑤^ | 0 | Low |
|  | Memory | Verbal learning test (2) | -1^①^ | 0 | 0 | -1^⑤^ | 0 | Low |
|  |  | Visual learning test (2) | -1^①^ | 0 | 0 | -1^⑤^ | 0 | Low |
|  |  | Forward visual span (2) | -1^①^ | 0 | 0 | -1^⑤^ | 0 | Low |
|  |  | Backward visual span (2) | -1^①^ | 0 | 0 | -1^⑤^ | 0 | Low |
|  |  | RMBT (2) | -1^①^ | 0 | 0 | -1^⑤^ | 0 | Low |
|  | Working memory | DSF (2) | -1^①^ | 0 | 0 | -1^⑤^ | 0 | Low |
|  |  | DSB (2) | -1^①^ | 0 | 0 | -1^⑤^ | 0 | Low |
|  |  | DST (2) | -1^①^ | 0 | 0 | -1^⑤^ | 0 | Low |
|  |  | TMT-A times (1) | -1^①^ | 0 | 0 | -1^⑤^ | 0 | Low |
|  |  | TMT-A errors (1) | -1^①^ | 0 | 0 | -1^⑤^ | 0 | Low |
|  | ADL | MBI (3) | -1^①^ | 0 | 0 | 0 | 0 | Moderate |
| Xu et al. (41) | Global cognition | MoCA (8) | -1^①^ | -2^③^ | 0 | 0 | -1^⑥^ | Very low |
|  |  | MoCA (1Hz) (2) | -1^①^ | 0 | 0 | -1^⑤^ | -1^⑥^ | Very low |
|  |  | MoCA (10Hz) (6) | -1^①^ | -2^③^ | 0 | 0 | -1^⑥^ | Very low |
|  | Memory | RBMT (7) | -1^①^ | -2^③^ | 0 | 0 | -1^⑥^ | Very low |
|  |  | RBMT (10Hz) (5) | -1^①^ | -2^③^ | 0 | 0 | -1^⑥^ | Very low |
|  | ADL | MBI (3) | -1^①^ | -2^③^ | 0 | -1^④^ | -1^⑥^ | Very low |
| Liu et al. (42) | Global cognition | MMSE (3) | -1^①^ | -2^③^ | 0 | -1^④⑤^ | 0 | Very low |
|  | Executive function | LOTCA (2) | -1^①^ | -2^③^ | 0 | -1^④⑤^ | 0 | Very low |
| Gong et al. (43) | Global cognition | MoCA (HF-rTMS) (2) | -1^①^ | 0 | 0 | -1^⑤^ | 0 | Low |
|  |  | MoCA (LF-rTMS) (2) | 0 | 0 | 0 | -1^⑤^ | 0 | Moderate |
|  |  | MMSE (HF-rTMS) (4) | -1^①^ | 0 | 0 | -1^⑤^ | 0 | Low |
|  |  | MMSE (LF-rTMS) (4) | -1^①^ | -2^③^ | 0 | -1^⑤^ | 0 | Very low |
|  | Executive function | LOTCA (2) | 0 | 0 | 0 | -1^⑤^ | 0 | Moderate |
|  | ADL | MBI (5) | -1^①^ | -2^③^ | 0 | -1^④⑤^ | 0 | Very low |
| Li et al. (44) | Global cognition | MoCA (6) | -1^①^ | 0 | 0 | 0 | -1^⑥^ | Low |
| Tian et al. (45) | Global cognition | MoCA (14) | -1^①^ | -1^②^ | 0 | 0 | 0 | Low |
|  |  | MMSE (6) | -1^①^ | 0 | 0 | 0 | 0 | Moderate |
|  | ADL | MBI (9) | -1^①^ | -2^③^ | 0 | 0 | 0 | Low |
|  | Executive function | LOTCA (4) | -1^①^ | 0 | 0 | 0 | 0 | Moderate |
|  | Cognitive deterioration | P300 latency (7) | -1^①^ | -2^③^ | 0 | -1^④^ | 0 | Very low |
|  |  | P300 amplitude (6) | -1^①^ | -2^③^ | 0 | 0 | 0 | Very low |
| Hara et al. (46) | Global cognition | MMSE, MoCA (3) | -1^①^ | 0 | 0 | 0 | -1^⑥^ | Low |
|  | Working memory | DSB, RBANS (3) | -1^①^ | 0 | 0 | 0 | -1^⑥^ | Low |
|  | Memory | RBMT (3) | -1^①^ | -1^②^ | 0 | 0 | -1^⑥^ | Very low |
|  | Attention | TMT-A (3) | -1^①^ | -2^③^ | 0 | 0 | -1^⑥^ | Very low |
| Chen et al. (47) | Global cognition | MoCA (19) | -1^①^ | -1^②^ | 0 | 0 | -1^⑥^ | Very low |
|  |  | MoCA (2-week duration)(6) | -1^①^ | 0 | 0 | 0 | -1^⑥^ | Low |
|  |  | MoCA (3-week duration) (6) | -1^①^ | 0 | 0 | 0 | -1^⑥^ | Low |
|  |  | MoCA (4-week duration) (23) | -1^①^ | -1^②^ | 0 | 0 | -1^⑥^ | Very low |
|  |  | MoCA (8-week duration) (7) | -1^①^ | 0 | 0 | 0 | -1^⑥^ | Low |
|  |  | MMSE (19) | -1^①^ | -2^③^ | 0 | 0 | -1^⑥^ | Very low |
|  |  | MMSE (2-week duration) (2) | -1^①^ | 0 | 0 | 0 | -1^⑥^ | Low |
|  |  | MMSE (3-week duration) (3) | -1^①^ | 0 | 0 | 0 | -1^⑥^ | Low |
|  |  | MMSE (4-week duration) (11) | -1^①^ | -2^③^ | 0 | 0 | -1^⑥^ | Very low |
|  |  | MMSE (8-week duration) (3) | -1^①^ | -2^③^ | 0 | 0 | -1^⑥^ | Very low |
|  |  | ADAS-Cog (3) | -1^①^ | 0 | 0 | 0 | -1^⑥^ | Low |
|  | Cognitive deterioration | P300 latency (15) | -1^①^ | -2^③^ | 0 | -1^④^ | -1^⑥^ | Very low |
|  |  | P300 latency (2-week duration) (2) | -1^①^ | -1^②^ | 0 | -1^④^ | -1^⑥^ | Very low |
|  |  | P300 latency (4-week duration) (9) | -1^①^ | -2^③^ | 0 | 0 | -1^⑥^ | Very low |
|  |  | P300 amplitude (15) | -1^①^ | -2^③^ | 0 | 0 | -1^⑥^ | Very low |
|  |  | P300 amplitude (2-week duration) (2) | -1^①^ | 0 | 0 | 0 | -1^⑥^ | Low |
|  |  | P300 amplitude (4-week duration) (9) | -1^①^ | -2^③^ | 0 | 0 | -1^⑥^ | Very low |
|  | Executive function | LOTCA (4) | -1^①^ | 0 | 0 | 0 | -1^⑥^ | Very low |

HF-rTMS, High frequency rTMS; LF-rTMS, Low frequency rTMS; -1 represents a downgrade; -2 represents two downgrade.

^①^ Risk of bias: In included studies with respect to randomization, blinding, allocation concealment, completeness of outcome data, or selective reporting risk of bias.

^②^ Inconsistency: There is moderate heterogeneity in the synthesis process of MAs/SRs data.

^③^ Inconsistency: High heterogeneity exists in the process of MA data synthesis.

^④^ Imprecision: The confidence interval of MA results is wide.

^⑤^ Imprecision: Part of the MAs/SRs sample size is small and the number of studies is small and the sample size is small.

^⑥^ Publication bias: Funnel plot shows asymmetry or no funnel plot is made.

^⑦^ Publication bias: Few studies were included and there may have been significant publication bias.
